# Supplementary material for: Effect of positive emotion intervention during late pregnancy on improving colostrum secretion: a randomised control trial protocol
Source: BMJ Open. 2023 Jun 30;13(6):e066601. doi: 10.1136/bmjopen-2022-066601 (PMC10314532; doi:10.1136/bmjopen-2022-066601)
Supplement: Author's manuscript [file bmjopen-2022-066601.draft_revisions.pdf]

BMJ Open is committed to open peer review. As part of this commitment we make the peer review history of every article we publish publicly available.

When an article is published we post the peer reviewers' comments and the authors' responses online. We also post the versions of the paper that were used during peer review. These are the versions that the peer review comments apply to.

The versions of the paper that follow are the versions that were submitted during the peer review process. They are not the versions of record or the final published versions. They should not be cited or distributed as the published version of this manuscript.

BMJ Open is an open access journal and the full, final, typeset and author-corrected version of record of the manuscript is available on our site with no access controls, subscription charges or pay-per-view fees (http://bmjopen.bmj.com).

If you have any questions on BMJ Open's open peer review process please email info.bmjopen@bmj.com

BMJ Open

The effect of positive emotion intervention during late pregnancy on improving colostrum secretion: a randomized control trial protocol

Journal:	*BMJ Open*
Manuscript ID	bmjopen-2022-066601
Article Type:	Protocol
Date Submitted by the Author:	12-Jul-2022

Complete List of Authors:	Xu, Kechen; Zhejiang University School of Medicine, School of Public Health, ; Wuyi First People's Hospital, Clinical laboratory center<br>Xie, Ying ; Zhejiang University School of Medicine<br>Han, Xiujun; Women's Hospital School of Medicine Zhejiang University<br>Yu, Ying; Women's Hospital School of Medicine Zhejiang University<br>Liu, Suqing; Wuyi First People's Hospital<br>Wu, Suliu; Wuyi First People's Hospital<br>Zhang, Qi; Old Dominion University, Community & Environmental Health<br>Yang, Qian; Zhejiang University - Zijingang Campus, public health

Keywords:	Neurobiology < NATURAL SCIENCE DISCIPLINES, PUBLIC HEALTH, PSYCHIATRY

SCHOLARONE™ Manuscripts

The effect of positive emotion intervention during late pregnancy on improving colostrum secretion: a randomized control trial protocol

Kechen Xu[1,3,a], Ying Xie[1,a], Xiujun Han[2,a], Ying Yu[2,a], Suqing Liu[3], Suliu Wu[3], Qi Zhang[4,*], Qian Yang[1,*]

1. Center for Health Policy Studies, School of Public Health and Department of Endocrinology, Children's Hospital, Zhejiang University School of Medicine, National Clinical Research Center for Child Health, Hangzhou, CN

2. Women's Hospital School of Medicine Zhejiang University, Hangzhou, Zhejiang, China

3. Wuyi First People's Hospital, Wuyi, Jinhua 321200, Zhejiang, China

4. Community & Environmental Health, Old Dominion University, Norfolk, Virginia, USA

a. These authors contributed equally to this article.

* Correspondence

Qian Yang [chianyoung@zju.edu.cn]

Qi Zhang [zhang@odu.edu]

For peer review only - http://bmjopen.bmj.com/site/about/guidelines.xhtml

ABSTRACT

Introduction Though evidence has revealed the beneficial effects of cognitive improvement interventions on breastfeeding, the effect of psychological interventions has rarely been studied. This study aims to test whether promoting a positive emotion intervention, “Three Good Things” intervention, during the last trimester of pregnancy can enhance early colostrum secretion and breastfeeding behaviors by modulating the hormones associated with lactation (prolactin and insulin-like growth factor Ⅰ). We will attempt to promote exclusive breastfeeding by using physiological behavioral measures.

Methods and analysis This study is designed as a randomized controlled trial conducted in the Women's Hospital School of Medicine at Zhejiang University and the Wuyi First People’s Hospital. The participants will be randomly divided into two groups using stratified random grouping: the intervention group will receive “Three Good Things” intervention while the control group will take corresponding contrast measures. These interventions will be continued from enrollment until the day of delivery. Maternal blood hormone levels will be tested approaching delivery and the following day after birth. Behavioral information about breastfeeding will be collected one week afterward.

Keywords Colostrum secretion; Positive psychology; Three good things; Breastfeeding; Randomized controlled trial.

Ethics and dissemination The study has been approved by the Ethics Committees of the Women's Hospital School of Medicine at Zhejiang University and the Wuyi First

For peer review only - http://bmjopen.bmj.com/site/about/guidelines.xhtml

People's Hospital. Results will be disseminated through peer-reviewed journals or international academic conferences.

Trial registration: Health management during pregnancy: research on breastfeeding promotion based on behavioural economics and positive psychological approaches, ChiCTR2000038849.

http://www.chictr.org.cn/usercenter.aspx

Strengths and limitations of this study

- Most previous breastfeeding-promotion interventions were performed in the postpartum period. This study moves the intervention period forward to the late stages of pregnancy.
- "Three Good Things", a typical psychological intervention will be used and evaluated for promoting positive emotions, which is feasible to carry out by individuals and easier to popularize.
- Our study attempts to promote breastfeeding by using positive psychological interventions to increase the hormones associated with lactation.
- This study attempts to explore the physiological mechanisms of the association between positive or negative emotions and breastfeeding behaviors.
- Strong hospital health education on breastfeeding may obscure the effects of this experimental intervention and lead to negative results.

For peer review only - http://bmjopen.bmj.com/site/about/guidelines.xhtml

Introduction

Breastfeeding is one of the most valid ways for promoting children's health and survival, which is advocated by the World Health Organization (WHO) and the United Nations Children's Fund (UNICEF)[1]. Breast milk, with its balanced nutrition, suitable temperature, and economic benefits, is considered the most ideal natural food for infants[2,3]. In addition, breast milk contains a diverse array of microbiota and myriad biologically-active components that contribute to the development of infants' immune systems. Thus, compared with formula-fed infants, breastfed children have fewer incidences of disease and lower mortality rates[4,5]. Breastfeeding also has economic benefits for individual families as well as at the national level by improving child development and reducing health costs[6]. As the WHO suggests, infants should be exclusively breastfed (given nothing else but breastmilk) for the first six months of life and be allowed to breast feed to 2 years of age or longer[7]. In 2012, the 56th World Health Assembly set a goal for 2025 to increase exclusive breastfeeding rates in the first six months to at least 50 percent[8]. However, few countries in the world fully meet the recommended standards, according to a recent report by UNICEF and the WHO in collaboration with the Global Breastfeeding Collective, a new initiative to increase global breastfeeding rates[8]. The *Global Breastfeeding Scorecard,* which evaluated 194 nations, found that only about 40% of children are breastfed exclusively within the first six months of life, and only 23 countries exceed the standard of 60% on their exclusive breastfeeding rates[8].

For peer review only

To promote exclusive breastfeeding in the first 6 months postpartum, a large number of studies have explored the influencing factors and intervention measures. A previous study indicated that a positive antenatal attitude toward breastfeeding was the strongest predictor and was associated with a 20-30% increase in breastfeeding initiation and maintenance at all time points[9]. Accordingly, the Breastfeeding Support Programme (BSP) was carried out to improve the attitudes and cognitions about breastfeeding among pregnant women. It was found to be an effective measure to delay the cessation of breastfeeding, increasing both its duration and its exclusivity[10].

In addition to cognitive factors, psychological status and emotions also play an important part in breastfeeding. Current evidence suggests that anxiety and depression are associated with lower exclusive breastfeeding behaviors[11–13]. Psychological distress resulted in low appetite, weight loss, and poor nutritional status, all of which made it difficult to breastfeed[14]. Emotional and traumatic stressors were found to have the greatest impact on breastfeeding outcomes[15]. Of clinical relevance, addressing prenatal and postpartum distress as a part of the implementation of breastfeeding practice interventions could improve breastfeeding rates[16]. Although intervention studies have been focusing on psychological distress, few studies have conducted positive psychology interventions to promote breastfeeding. Positive psychology interventions (PPIs) can enhance women's mental well-being during the perinatal period[17]. Thus, PPIs may be worth exploring as measures to promote breastfeeding behaviors[18]. The "Three Good Things", as a PPI instructs participants to write down three good things every night, was proposed by Seligman et al. in 2005. It helps to develop skills in

For peer review only - http://bmjopen.bmj.com/site/about/guidelines.xhtml

regulating emotions[22], which can enhance the experience of positive emotions and alleviate the symptoms of depression[23]. This psychological intervention has been tested in multiple studies and is replicable and easy to implement by individuals. Therefore, this study will use and evaluate the effect of "Three Good Things" as an intervention to enhance positive emotions and reduce negative emotions among pregnant women.

Breastfeeding behaviors are influenced by lactation capacity, which is reflected in hormone levels. Prolactin (PRL) is a protein hormone secreted by the anterior pituitary gland that can stimulate and maintain lactation. Insulin-like growth factor I (IGF-I) is a kind of polypeptide that can mediate the galactagogue of growth hormone. Previous studies have shown that emotions and the secretion of these two hormones are closely related. Positive emotions can promote the secretion of prolactin, while depression can lead to a prolactin secretion imbalance, and stress events and anxiety can also lead to the low secretion level of IGF-I[19–21]. This study aims to use “Three Good Things” intervention to promote positive emotions among pregnant woman during mid- and late pregnancy, thus improving the secretion of lactation-related hormones and the initiation and continuation of breastfeeding behaviors.

For peer review only - http://bmjopen.bmj.com/site/about/guidelines.xhtml

Methods and analysis

Aims

The study is designed as a randomized controlled trial in China, to test whether the "Three Good Things" intervention during late pregnancy can enhance colostrum secretion and related breastfeeding behaviors and effects. We also aim to examine whether the association between prenatal emotions and postnatal breastfeeding behaviors are mediated by lactation-related hormones.

Hypotheses

Hypotheses 1a: "Three Good Things" interventions can promote the secretion of PRL and IGF-I by enhancing positive emotions and reducing negative emotions of pregnant women.

Hypotheses 1b: "Three Good Things" interventions can improve maternal breastfeeding willingness and postpartum in-hospital breastfeeding behaviors by enhancing positive emotions and reducing negative emotions.

Hypotheses 2a: Increased secretion of PRL and IGF-I will predict promotion in breastfeeding behaviors and effect.

Hypotheses 2b: Positive emotions can indirectly promote breastfeeding behaviors during postpartum hospitalization through the secretion of PRL and IGF-I, while negative emotions have adverse effects.

Study Design

This is the protocol for a randomized controlled clinical trial. Between October 2020 and June 2021, a pilot study was carried out at the First People's Hospital of Wuyi

For peer review only - http://bmjopen.bmj.com/site/about/guidelines.xhtml

County in Zhejiang Province. During this time, a total of 96 pregnant women were recruited for the test of the designed survey questionnaire, including baseline survey and follow-up survey, and the intervention questionnaire. In January 2022, the formal trial was conducted in Women's Hospital School of Medicine at Zhejiang University. This study will have three phases: T0, baseline; T1, immediate postintervention; T2, follow-up (one week after delivery). The qualitative and quantitative data will be collected in the three phases. T1 is a baseline survey collecting demographic information, data about existing positive and negative emotions, and their intention to breastfeed. T2 is the intervention process for the intervention and the control groups, with process variables of emotion and willingness collected every week. T3 is the pre- and post-natal hospitalization period when we will collect outcome indicators such as serum hormone levels, positive and negative emotions, intention to breastfeed, breastfeeding behavior, and other indicators. Figure 1 illustrates the overall study design.

Recruitment

The study will be conducted in the Women's Hospital School of Medicine at Zhejiang University. We will include primiparous pregnant women at 34 to 38 gestational weeks. The exclusion criteria are as follows:

- Women with no intention to give birth in a designated hospital.
- Women not giving birth for the first time.
- Women with a disease that prevents breastfeeding (cardiac insufficiency, infectious diseases, neurological or cognitive damage, et al.)

For peer review only - http://bmjopen.bmj.com/site/about/guidelines.xhtml

- Pregnant women with more than one fetus.

Sample size

Based on the results of the pilot study, statistical power (1-β) of 80%, and a two-tailed significance level (α) of 0.05, we calculated the targeted sample size with a two-sample t-test allowing unequal variance. Given the breastfeeding behavior score (LATCH) was 12.72 in the intervention group (standard deviation, SD = 2.33) and 12.06 in the control group (SD = 1.51), the theoretical sample size was shown as N1 = N2 = 141. Considering a dropout rate of 20%, the ultimate sample size needed was estimated at 353 individuals.

Randomization and blinding

Medical staff at the hospital's fetal heart monitoring studio will help recruit participants. We will use stratified random grouping to divide the pregnant women into two groups according to the level of support they say they receive (high, average, none) from family members in the oral interview. Pregnant women received on the same day will be randomly assigned to the intervention group and the control group according to the level of support (high, average, none). Our trial will be a single-blinded trial of participants.

Intervention

At fetal heart monitoring studio, we will ask the participants to add our Wechat account, and the subsequent communications and relevant questionnaire distribution will be carried out through Wechat chat software. In the T1 stage, participants will complete a series of intervention questionnaires. Participants in the intervention group

For peer review only - http://bmjopen.bmj.com/site/about/guidelines.xhtml

will be asked to write down three good things that have happened during the day every two days, with weekly specific themes. Participants in the control group will only have to write about three things that come to mind first that are related to the same theme. To improve the compliance and interest of the participants, we will set up different instructions and themes for them weekly (Table 1). Meanwhile, if a participant forgets to write, we will send out hints if they fail to complete the questionnaires. When the participant is unwilling to take the initiative to fill in the questionnaire, we will ask for reasons and try to encourage her to continue to participate. If she gives up, we will keep the corresponding survey data, recorded as loss to follow up in the database.

Evaluation and outcomes

Primary outcomes will include exclusive breastfeeding and on-demand feeding during postnatal hospitalization. Exclusive breastfeeding means that the baby is not given any liquid or solid food other than breast milk. On-demand feeding means breastfeeding according to the baby's needs. Since the infant's early urination reflects breastfeeding, changing diapers 6-8 times a day in the early postpartum period is considered feeding on demand.

Secondary outcome indicators will mainly include other breastfeeding behaviors during postpartum hospitalization (first postnatal food for babies is breast milk; LATCH breastfeeding score), lactation-related hormone secretion (prolactin and IGF-I), and postpartum breastfeeding intention and postpartum emotion.

Data collection and follow-up

For peer review only - http://bmjopen.bmj.com/site/about/guidelines.xhtml

On the day of enrollment, pregnant women will be asked to complete an enrollment questionnaire providing information about general demographic characteristics and other factors influencing breastfeeding. Meanwhile, if the enrollment questionnaire finds prenatal depression among the participants, we will refer them to the department of psychology. Then ecological instantaneous evaluation of both groups will be performed every week to determine the trend of change in intention and emotions until delivery. The measurement of emotion uses the Positive and Negative Affect Scale (PANAS) and 10 positive items for positive emotions proposed by Barbara L. Fredrickson for validation[24,25]. Postpartum questionnaires on breastfeeding behavior during postnatal hospitalization will be completed one week after delivery. Blood samples will be collected twice, approaching delivery and on the second day after delivery, and evaluated for prolactin and IGF-I to evaluate postpartum lactation ability.

Upon recruitment, general demographic variables will be collected: education, occupation, number of permanent household members, total average annual household income, and expected date of birth. Other influencing factors will include mode of delivery, self-reported ease of breastfeeding, degree of sleep disturbance, self-reported importance of early exclusive breastfeeding, knowledge of breastfeeding, birth injury and nipple cracking pain scores, degree of hospital support, depression (measured by Edinburgh Postnatal Depression Scale, EPDS), anxiety (measured by Generalized Anxiety Disorder, GAD-7), breastfeeding intentions (measured by Infant Feeding Intentions Scale, IFI), breastfeeding behavior score (LATCH), resilience (Brief Resilience Scale, BRS), and potential number of traumatic events[26,27(p7),28–31].

For peer review only - http://bmjopen.bmj.com/site/about/guidelines.xhtml

All questionnaires will be made through the questionnaire star website and distributed via WeChat. The corresponding data are stored in the questionnaire star account. Hormone test results will be recorded and saved manually. We will keep the personal information and data of each participant strictly confidential.

Data analysis

Descriptive analyses will be performed to show the baseline characteristics of the participants. To assess the intervention effects over time, MANOVA of repeated measuring will be taken for the change of positive and negative emotions, intention to breastfeed, and resilience trend across the weeks. To test the association among positive/negative emotions, lactation-related hormones, and breastfeeding behaviors, we will perform multiple linear regression or logistic regression in between and exploratory mediation analysis. To evaluate the effect of the intervention, we will take a two-sample T-test or Wilcoxon rank-sum test for quantitative data and a Chi-square test for qualitative data.

Data monitoring

The data monitoring committee is composed of members of the science and education Department of the hospital. As the administrative department of the hospital, the Ministry of Science and Education is in charge of scientific research, and reviews the integrity of scientific research to ensure the authenticity of data.

Discussion

Our study has several strengths. First, as distinct from current interventions focusing on knowledge improvement, our study attempts to promote secretion of lactation-related

For peer review only - http://bmjopen.bmj.com/site/about/guidelines.xhtml

hormones by elevating positive emotions, then breastfeeding behaviors. Second, most previous interventions promoting breastfeeding were set in the postpartum period. Considering the importance of early breastfeeding experiences, we moved the intervention period to the late stages of pregnancy. Third, earlier mixed educational and supportive interventions are difficult to evaluate and replicate precisely. Our study will use the typical psychological measures, “Three Good Things”, to boost positive emotions, which is feasible for individuals to carry out and can be easier to promote and popularize among pregnant women.

This study also has several limitations. First, since our program is conducted in a maternity hospital with well-developed breastfeeding courses, differences among groups may be hard to detect due to the generally high level of breastfeeding knowledge. Second, due to the restrictions of the testing technology and the intricacies involved in distinguishing between endogenous and exogenous sources, we will not test for oxytocin in the serum, although this has been widely reported to be associated with lactation. However, oxytocin release is related to prolactin levels, which will be included in our test[32]. Third, we limit the outcome measurement of breastfeeding-related behaviors to the postpartum hospitalization period, without following a 6-month or longer period. Nevertheless, exclusive breastfeeding in the early postpartum period (1 hour and within a few days after delivery) has been positively correlated with the duration of breastfeeding after delivery.

In conclusion, this study will test the effect of “Three Good Things” intervention not only on emotions of women during pregnancy, but also on the secretion of

For peer review only - http://bmjopen.bmj.com/site/about/guidelines.xhtml

colostrum and breastfeeding behaviors as well. This study will also help us further identify the physiological mechanisms of the association between emotions and breastfeeding behaviors.

List of abbreviations

WHO	(World Health Organization)
UNICEF	(United Nations Children's Fund)
IGF-I	(Insulin-like growth factor Ⅰ)
PRL	(Prolactin)
PANAS	(Positive and Negative Affect Scale)
EPDS	(Edinburgh Postnatal Depression Scale)
GAD-7	(Generalized Anxiety Disorder-7)
IFI	(Infant Feeding Intentions Scale)
BRS	(Brief Resilience Scale)

Ethics and dissemination

The study has been approved by the Ethics Committees of the Women's Hospital School of Medicine at Zhejiang University (20190088) and the Wuyi First People's Hospital. The study will be conducted by the Declaration of Helsinki ethical principles for research involving human subjects. Results will be disseminated through peer-reviewed journals or international academic conferences. Written consent will be obtained from all participants.

References

1. Infant and young child feeding. Accessed December 27, 2021.

For peer review only - http://bmjopen.bmj.com/site/about/guidelines.xhtml

https://www.who.int/en/news-room/fact-sheets/detail/infant-and-young-child-feeding

2. Andreas NJ, Kampmann B, Mehring Le-Doare K. Human breast milk: A review on its composition and bioactivity. *Early Hum Dev*. 2015;91(11):629-635. doi:10.1016/j.earlhumdev.2015.08.013

3. Johnson TJ, Patel AL, Bigger HR, Engstrom JL, Meier PP. Economic benefits and costs of human milk feedings: a strategy to reduce the risk of prematurity-related morbidities in very-low-birth-weight infants. *Adv Nutr Bethesda Md*. 2014;5(2):207-212. doi:10.3945/an.113.004788

4. Le Doare K, Holder B, Bassett A, Pannaraj PS. Mother's Milk: A Purposeful Contribution to the Development of the Infant Microbiota and Immunity. *Front Immunol*. 2018;9:361. doi:10.3389/fimmu.2018.00361

5. Lyons KE, Ryan CA, Dempsey EM, Ross RP, Stanton C. Breast Milk, a Source of Beneficial Microbes and Associated Benefits for Infant Health. *Nutrients*. 2020;12(4):E1039. doi:10.3390/nu12041039

6. The Lancet Breastfeeding Series papers . 2016.

7. Global strategy for infant and young child feeding,WHO.

8. Babies and mothers worldwide failed by lack of investment in breastfeeding. Accessed December 27, 2021. https://www.who.int/news/item/01-08-2017-babies-and-mothers-worldwide-failed-by-lack-of-investment-in-breastfeeding

9. Amiel Castro RT, Glover V, Ehlert U, O'Connor TG. Antenatal psychological and socioeconomic predictors of breastfeeding in a large community sample. *Early Hum Dev*. 2017;110:50-56. doi:10.1016/j.earlhumdev.2017.04.010

10. van Dellen SA, Wisse B, Mobach MP, Dijkstra A. The effect of a breastfeeding support programme on breastfeeding duration and exclusivity: a quasi-experiment. *BMC Public Health*. 2019;19(1):993. doi:10.1186/s12889-019-7331-y

For peer review only - http://bmjopen.bmj.com/site/about/guidelines.xhtml

11. Ystrom E. Breastfeeding cessation and symptoms of anxiety and depression: a longitudinal cohort study. *BMC Pregnancy Childbirth*. 2012;12:36. doi:10.1186/1471-2393-12-36

12. Seimyr L, Edhborg M, Lundh W, Sjögren B. In the shadow of maternal depressed mood: experiences of parenthood during the first year after childbirth. *J Psychosom Obstet Gynaecol*. 2004;25(1):23-34. doi:10.1080/01674820410001737414

13. Kehler HL, Chaput KH, Tough SC. Risk factors for cessation of breastfeeding prior to six months postpartum among a community sample of women in Calgary, Alberta. *Can J Public Health Rev Can Sante Publique*. 2009;100(5):376-380.

14. Emerson JA, Tol W, Caulfield LE, Doocy S. Maternal Psychological Distress and Perceived Impact on Child Feeding Practices in South Kivu, DR Congo. *Food Nutr Bull*. 2017;38(3):319-337. doi:10.1177/0379572117714385

15. Kitsantas P, Gaffney KF, Nirmalraj L, Sari M. The influence of maternal life stressors on breastfeeding outcomes: a US population-based study. *J Matern-Fetal Neonatal Med Off J Eur Assoc Perinat Med Fed Asia Ocean Perinat Soc Int Soc Perinat Obstet*. 2019;32(11):1869-1873. doi:10.1080/14767058.2017.1421929

16. Ritchie-Ewing G, Mitchell AM, Christian LM. Associations of Maternal Beliefs and Distress in Pregnancy and Postpartum With Breastfeeding Initiation and Early Cessation. *J Hum Lact Off J Int Lact Consult Assoc*. 2019;35(1):49-58. doi:10.1177/0890334418767832

17. Corno G, Espinoza M, Maria Baños R. A narrative review of positive psychology interventions for women during the perinatal period. *J Obstet Gynaecol J Inst Obstet Gynaecol*. 2019;39(7):889-895. doi:10.1080/01443615.2019.1581735

18. Shariat M, Abedinia N. The Effect of Psychological Intervention on Mother-Infant Bonding and Breastfeeding. *Iran J Neonatol*. 2017;8(1):7-15.

For peer review only - http://bmjopen.bmj.com/site/about/guidelines.xhtml

doi:10.22038/ijn.2017.16673.1191

19. Turner RA, Altemus M, Yip DN, et al. Effects of emotion on oxytocin, prolactin, and ACTH in women. *Stress Amst Neth*. 2002;5(4):269-276. doi:10.1080/1025389021000037586-1

20. Nicholas L, Dawkins K, Golden RN. Psychoneuroendocrinology of depression. Prolactin. *Psychiatr Clin North Am*. 1998;21(2):341-358. doi:10.1016/s0193-953x(05)70009-5

21. Field T. Prenatal anxiety effects: A review. *Infant Behav Dev*. 2017;49:120-128. doi:10.1016/j.infbeh.2017.08.008

22. Seligman MEP, Steen TA, Park N, Peterson C. Positive psychology progress: empirical validation of interventions. *Am Psychol*. 2005;60(5):410-421. doi:10.1037/0003-066X.60.5.410

23. Beanlands H, McCay E, Fredericks S, et al. Decreasing stress and supporting emotional well-being among senior nursing students: A pilot test of an evidence-based intervention. *Nurse Educ Today*. 2019;76:222-227. doi:10.1016/j.nedt.2019.02.009

24. Watson D, Clark LA, Tellegen A. Development and validation of brief measures of positive and negative affect: the PANAS scales. *J Pers Soc Psychol*. 1988;54(6):1063-1070. doi:10.1037//0022-3514.54.6.1063

25. Fredrickson BL. Positive Emotions Broaden and Build. In: Devine P, Plant A, eds. *Advances in Experimental Social Psychology, Vol 47*. Vol 47. Elsevier Academic Press Inc; 2013:1-53. doi:10.1016/B978-0-12-407236-7.00001-2

26. Cox JL, Holden JM, Sagovsky R. Detection of postnatal depression. Development of the 10-item Edinburgh Postnatal Depression Scale. *Br J Psychiatry J Ment Sci*. 1987;150:782-786. doi:10.1192/bjp.150.6.782

27. Spitzer RL, Kroenke K, Williams JBW, Löwe B. A brief measure for assessing

For peer review only

For peer review only - http://bmjopen.bmj.com/site/about/guidelines.xhtml

generalized anxiety disorder: the GAD-7. *Arch Intern Med*. 2006;166(10):1092-1097. doi:10.1001/archinte.166.10.1092

28. Nommsen-Rivers LA, Dewey KG. Development and validation of the infant feeding intentions scale. *Matern Child Health J*. 2009;13(3):334-342. doi:10.1007/s10995-008-0356-y

29. Jensen D, Wallace S, Kelsay P. LATCH: a breastfeeding charting system and documentation tool. *J Obstet Gynecol Neonatal Nurs JOGNN*. 1994;23(1):27-32. doi:10.1111/j.1552-6909.1994.tb01847.x

30. Smith BW, Dalen J, Wiggins K, Tooley E, Christopher P, Bernard J. The brief resilience scale: assessing the ability to bounce back. *Int J Behav Med*. 2008;15(3):194-200. doi:10.1080/10705500802222972

31. Peterson C, Park N, Pole N, D'Andrea W, Seligman MEP. Strengths of character and posttraumatic growth. *J Trauma Stress*. 2008;21(2):214-217. doi:10.1002/jts.20332

32. Uvnäs Moberg K, Ekström-Bergström A, Buckley S, et al. Maternal plasma levels of oxytocin during breastfeeding-A systematic review. *PloS One*. 2020;15(8):e0235806. doi:10.1371/journal.pone.0235806

Footnotes

Availability of data and materials

Not applicable.

Authors' contributions

KCX, YX, XJH and YY contributed to data acquisition and analysis. KCX and YX were in charge of the writing. SQL and SLW participated in the implementation of the

For peer review only - http://bmjopen.bmj.com/site/about/guidelines.xhtml

pilot trial. QY and QZ contributed to study design and manuscript revision. All authors approved the final version of the manuscript.

Funding statement

This study is supported by the National Natural Science Foundation of China (71974170), Medical Scientific Research Foundation of Zhejiang Province of China (2021455676). The funders have no influences on study methods, analysis, interpretation, or publication of results.

Competing interests statement

The authors declare no conflict of interest. The funders have no influences on study methods, analysis, interpretation, or publication of results.

Patient consent for publication

Not required.

Acknowledgements

We appreciate the support of the clinical staff of Zhejiang University School of Medicine and Wuyi First People's Hospital, including Xinyi Zhou, Suqing Liu, Xiaohong Qin, Qin Geng, and Di Gu.

Figure 1. Flow chart of the breastfeeding intervention project.The participants will be randomly divided into intervention group and control group. In phase T0, participants will complete the first questionnaire, including breastfeed intention, emotional scale, and writing practice persistence. In the T1 stage, the participants will complete a series of intervention questionnaires, including breastfeed intention, emotional scale, writing practice persistence. First lactation hormone test will be performed before delivery. The second lactation hormone test will be performed after delivery. Finally, a postpartum questionnaire will be performed about the outcome of lactation behavior.

For peer review only

431 **Table 1.** Instructions for the Intervention and Control Groups.

For peer review only

For peer review only - http://bmjopen.bmj.com/site/about/guidelines.xhtml

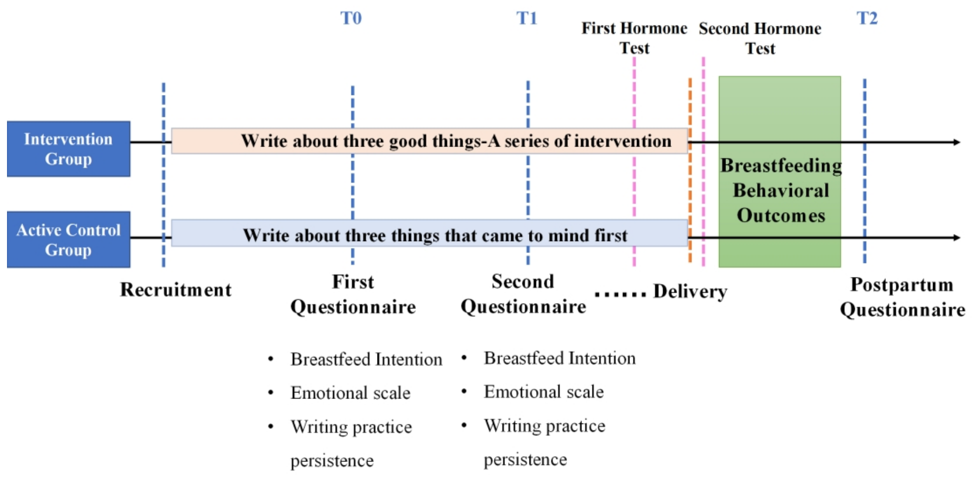

338x190mm (192 x 192 DPI)

Table 1 The instructions for the intervention and control group

Week	Topic	Instructions for the intervention group	Instructions for the control group
**1**	**My baby**	Please recall 3 good things that happened to you today related to you and your baby, and record them with the reasons why they happened. Here are some examples:<br>(1) My baby is moving today, and my baby is growing healthily.<br>(2) I saw a cute baby picture today, and my baby may be cuter.<br>(3) I bought baby's little clothes today, they are so small and cute.	Please record the first three baby-related events that come to your mind today, and record them and the reasons why they happened. Here are some examples:<br>(1) I suddenly thought that it is not easy to have a child.<br>(2) I had the maternity checkup today.
**2**	**Nature and me**	Please recall 3 good things that happened to you today related to nature, and record them with the reasons why they happened. Here are some examples:<br>(1) I heard a bird chirping on the road today, is it saying hello to me?<br>(2) The small flower we planted is blooming, we have taken good care of it.<br>(3) The sky is blue today, and there are traces of airplanes flying over.	Please record the first three nature-related events that come to your mind today, and record them and the reasons why they happened. Here are some examples:<br>(1) It's raining today, and the air is wet.<br>(2) I watered the flowers at home.
**3**	**My family**	Please recall 3 good things that happened to you today related to your family, and record them with the reasons why they happened. Here are some examples:<br>(1) My husband is very considerate, and I feel very happy.<br>(2) My mother and mother-in-law specially cook duck for me, they always love me very much.<br>(3) All the people I love and I am expecting the baby together. My friends also told me just take it easy and not to be nervous.	Please record the first three family-related events that come to your mind today, and record them and the reasons why they happened. Here are some examples:<br>(1) My husband and I had dinner with our parents today.<br>(2) A good friend of mine got married today.
**4**	**My future**	Please recall 3 good things that happened to you today related to the future, and record them with the reasons why they happened. Here are some examples:<br>(1) Today, I saw other people's little kids, this time next year I can have my own baby!<br>(2) The weather forecast says the weather will be good, I'm looking forward to sunshine tomorrow.	Please record the first three future-related events that come to your mind today, and record them and the reasons why they happened. Here are some examples:<br>(1) We're going for a checkup tomorrow.<br>(2) Tomorrow my mother-in-law is coming to visit us.

For peer review only

		(3) Tomorrow my favorite TV series will be updated.	
**5**	**My life**	Please recall 3 good things that happened to you today related to your life, and record them with the reasons why they happened. Here are some examples:<br>(1) Today, I listened to my favorite songs, which gave me a lot of inspiration and memories.<br>(2) Today, I found a gentle sentence on my phone, which said, "I was born mediocre, and I was born proud. Let's be a happy ordinary person. "<br>(3) The emoticons my friend sent me today were so cute, I thought they were very interesting and funny.	Please record the first three life-related events that come to your mind today, and record them and the reasons why they happened. Here are some examples:<br>(1) I watch videos on the phone for an hour today.<br>(2) I watched a TV series with my husband this evening.

For peer review only

SPIRIT

STANDARD PROTOCOL ITEMS: RECOMMENDATIONS FOR INTERVENTIONAL TRIALS

SPIRIT 2013 Checklist: Recommended items to address in a clinical trial protocol and related documents*

Section/item	ItemNo	Description	
**Administrative information**			
Title	1	Descriptive title identifying the study design, population, interventions, and, if applicable, trial acronym	Title, Page 1
Trial registration	2a	Trial identifier and registry name. If not yet registered, name of intended registry	Trial registration, Page 2
	2b	All items from the World Health Organization Trial Registration Data Set	Trial registration, Page 2
Protocol version	3	Date and version identifier	NA
Funding	4	Sources and types of financial, material, and other support	Funding, Page14
Roles and responsibilities	5a	Names, affiliations, and roles of protocol contributors	Authors' contributions, Page14
	5b	Name and contact information for the trial sponsor	Correspondence, Page1
	5c	Role of study sponsor and funders, if any, in study design; collection, management, analysis, and interpretation of data; writing of the report; and the decision to submit the report for publication, including whether they will have ultimate authority over any of these activities	Competing interests, Page14
	5d	Composition, roles, and responsibilities of the coordinating centre, steering committee, endpoint adjudication committee, data management team, and other individuals or groups overseeing the trial, if applicable (see Item 21a for data monitoring committee)	NA
**Introduction**			
Background and rationale	6a	Description of research question and justification for undertaking the trial, including summary of relevant studies (published and unpublished) examining benefits and harms for each intervention	Background, paragraph 2-3
	6b	Explanation for choice of comparators	NA

For peer review only

For peer review only - http://bmjopen.bmj.com/site/about/guidelines.xhtml

Objectives	7	Specific objectives or hypotheses	Background, paragraph 4
Trial design	8	Description of trial design including type of trial (eg, parallel group, crossover, factorial, single group), allocation ratio, and framework (eg, superiority, equivalence, noninferiority, exploratory)	Methods/Design, paragraph 4

Methods: Participants, interventions, and outcomes

Study setting	9	Description of study settings (eg, community clinic, academic hospital) and list of countries where data will be collected. Reference to where list of study sites can be obtained	Study Design, Page 7-8
Eligibility criteria	10	Inclusion and exclusion criteria for participants. If applicable, eligibility criteria for study centres and individuals who will perform the interventions (eg, surgeons, psychotherapists)	Recruitment, Page 8
Interventions	11a	Interventions for each group with sufficient detail to allow replication, including how and when they will be administered	Intervention, Page 9-10
	11b	Criteria for discontinuing or modifying allocated interventions for a given trial participant (eg, drug dose change in response to harms, participant request, or improving/worsening disease)	NA
	11c	Strategies to improve adherence to intervention protocols, and any procedures for monitoring adherence (eg, drug tablet return, laboratory tests)	Intervention, Page 9-10
	11d	Relevant concomitant care and interventions that are permitted or prohibited during the trial	NA
Outcomes	12	Primary, secondary, and other outcomes, including the specific measurement variable (eg, systolic blood pressure), analysis metric (eg, change from baseline, final value, time to event), method of aggregation (eg, median, proportion), and time point for each outcome. Explanation of the clinical relevance of chosen efficacy and harm outcomes is strongly recommended	Evaluation and outcomes, Page 10
Participant timeline	13	Time schedule of enrolment, interventions (including any run-ins and washouts), assessments, and visits for participants. A schematic diagram is highly recommended (see Figure)	Data collection and follow-up, Page 10-11

For peer review only - http://bmjopen.bmj.com/site/about/guidelines.xhtml

Sample size	14	Estimated number of participants needed to achieve study objectives and how it was determined, including clinical and statistical assumptions supporting any sample size calculations	Sample size, Page 9
Recruitment	15	Strategies for achieving adequate participant enrolment to reach target sample size	Randomization and blinding, Page 9
**Methods: Assignment of interventions (for controlled trials)**			
Allocation:			
Sequence generation	16a	Method of generating the allocation sequence (eg, computer-generated random numbers), and list of any factors for stratification. To reduce predictability of a random sequence, details of any planned restriction (eg, blocking) should be provided in a separate document that is unavailable to those who enrol participants or assign interventions	Randomization and blinding, Page 9
Allocation concealme nt mechanism	16b	Mechanism of implementing the allocation sequence (eg, central telephone; sequentially numbered, opaque, sealed envelopes), describing any steps to conceal the sequence until interventions are assigned	Randomization and blinding, Page 9
Implementa tion	16c	Who will generate the allocation sequence, who will enrol participants, and who will assign participants to interventions	Randomization and blinding, Page 9
Blinding (masking)	17a	Who will be blinded after assignment to interventions (eg, trial participants, care providers, outcome assessors, data analysts), and how	Randomization and blinding, Page 9
	17b	If blinded, circumstances under which unblinding is permissible, and procedure for revealing a participant's allocated intervention during the trial	NA
**Methods: Data collection, management, and analysis**			
Data collection methods	18a	Plans for assessment and collection of outcome, baseline, and other trial data, including any related processes to promote data quality (eg, duplicate measurements, training of assessors) and a description of study instruments (eg, questionnaires, laboratory tests) along with their reliability and validity, if known. Reference to where data collection forms can be found, if not in the protocol	Data collection and follow-up, Page 10-11
	18b	Plans to promote participant retention and complete follow-up, including list of any outcome data to be collected for participants who discontinue or deviate from intervention protocols	Intervention, Page 9-10

Data management	19	Plans for data entry, coding, security, and storage, including any related processes to promote data quality (eg, double data entry; range checks for data values). Reference to where details of data management procedures can be found, if not in the protocol	Data collection and follow-up, Page 10-11
Statistical methods	20a	Statistical methods for analysing primary and secondary outcomes. Reference to where other details of the statistical analysis plan can be found, if not in the protocol	Data analysis, Page 11-12
	20b	Methods for any additional analyses (eg, subgroup and adjusted analyses)	NA
	20c	Definition of analysis population relating to protocol non-adherence (eg, as randomised analysis), and any statistical methods to handle missing data (eg, multiple imputation)	NA
**Methods: Monitoring**			
Data monitoring	21a	Composition of data monitoring committee (DMC); summary of its role and reporting structure; statement of whether it is independent from the sponsor and competing interests; and reference to where further details about its charter can be found, if not in the protocol. Alternatively, an explanation of why a DMC is not needed	Data monitoring, Page 12
	21b	Description of any interim analyses and stopping guidelines, including who will have access to these interim results and make the final decision to terminate the trial	NA
Harms	22	Plans for collecting, assessing, reporting, and managing solicited and spontaneously reported adverse events and other unintended effects of trial interventions or trial conduct	NA
Auditing	23	Frequency and procedures for auditing trial conduct, if any, and whether the process will be independent from investigators and the sponsor	NA
**Ethics and dissemination**			
Research ethics approval	24	Plans for seeking research ethics committee/institutional review board (REC/IRB) approval	Ethics approval and consent to participate, Page 14
Protocol amendments	25	Plans for communicating important protocol modifications (eg, changes to eligibility criteria, outcomes, analyses) to relevant parties (eg, investigators, REC/IRBs, trial participants, trial registries, journals, regulators)	NA

For peer review only - http://bmjopen.bmj.com/site/about/guidelines.xhtml

Consent or assent	26a	Who will obtain informed consent or assent from potential trial participants or authorised surrogates, and how (see Item 32)	Ethics approval and consent to participate, Page 14
	26b	Additional consent provisions for collection and use of participant data and biological specimens in ancillary studies, if applicable	NA
Confidentiality	27	How personal information about potential and enrolled participants will be collected, shared, and maintained in order to protect confidentiality before, during, and after the trial	Intervention, Data collection and follow-up, Page 9-11
Declaration of interests	28	Financial and other competing interests for principal investigators for the overall trial and each study site	Competing interests, Page 14
Access to data	29	Statement of who will have access to the final trial dataset, and disclosure of contractual agreements that limit such access for investigators	NA
Ancillary and post-trial care	30	Provisions, if any, for ancillary and post-trial care, and for compensation to those who suffer harm from trial participation	NA
Dissemination policy	31a	Plans for investigators and sponsor to communicate trial results to participants, healthcare professionals, the public, and other relevant groups (eg, via publication, reporting in results databases, or other data sharing arrangements), including any publication restrictions	NA
	31b	Authorship eligibility guidelines and any intended use of professional writers	NA
	31c	Plans, if any, for granting public access to the full protocol, participant-level dataset, and statistical code	NA
**Appendices**			
Informed consent materials	32	Model consent form and other related documentation given to participants and authorised surrogates	Informed consent materials
Biological specimens	33	Plans for collection, laboratory evaluation, and storage of biological specimens for genetic or molecular analysis in the current trial and for future use in ancillary studies, if applicable	NA

*It is strongly recommended that this checklist be read in conjunction with the SPIRIT 2013 Explanation & Elaboration for important clarification on the items. Amendments to the protocol should be tracked and dated. The SPIRIT checklist is copyrighted by the SPIRIT

For peer review only

Group under the Creative Commons "Attribution-NonCommercial-NoDerivs 3.0 Unported" license.

For peer review only

For peer review only - http://bmjopen.bmj.com/site/about/guidelines.xhtml

BMJ Open

The effect of positive emotion intervention during late pregnancy on improving colostrum secretion: a randomized control trial protocol

Journal:	*BMJ Open*
Manuscript ID	bmjopen-2022-066601.R1
Article Type:	Protocol
Date Submitted by the Author:	08-Mar-2023
Complete List of Authors:	Xu, Kechen; Zhejiang University School of Medicine, School of Public Health, ; Wuyi First People's Hospital, Clinical laboratory center<br>Xie, Ying ; Chinese Academy of Medical Sciences & Peking Union Medical College, School of Health Policy and Management<br>Han, Xiujun; Women's Hospital School of Medicine Zhejiang University<br>Yu, Ying; Women's Hospital School of Medicine Zhejiang University<br>Liu, Suqing; Wuyi First People's Hospital<br>Wu, Suliu; Wuyi First People's Hospital<br>Yang, Qian; Zhejiang University School of Public Health, Center for Health Policy Studies; The Fourth Affiliated Hospital Zhejiang University School of Medicine, the department of Geriatrics<br>Zhang, Qi; Old Dominion University, Community & Environmental Health
**Primary Subject Heading**:	Obstetrics and gynaecology
Secondary Subject Heading:	Public health, Mental health
Keywords:	PUBLIC HEALTH, PSYCHIATRY, OBSTETRICS

SCHOLARONE™
Manuscripts

The effect of positive emotion intervention during late pregnancy on improving colostrum secretion: a randomized control trial protocol

Kechen Xu[1,2,a], Ying Xie[3,a], Xiujun Han[4,a], Ying Yu[4,a], Suqing Liu[2], Suliu Wu[2], Qian Yang[1,*], Qi Zhang[5]

1. School of Public Health and the department of Geriatrics, the Fourth Affiliated Hospital, Zhejiang University School of Medicine, Zhejiang University, Zhejiang University, Hangzhou, 310058, China, CN

2. Wuyi First People's Hospital, Wuyi, Jinhua 321200, Zhejiang, China

3. School of Health Policy and Management, Chinese Academy of Medical Sciences and Peking Union Medical College, Beijing, China

4. Women's Hospital School of Medicine Zhejiang University, Hangzhou, Zhejiang, China

5. Community & Environmental Health, Old Dominion University, Norfolk, Virginia, USA

a. These authors contributed equally to this article.

* Correspondence

Qian Yang [chianyoung@zju.edu.cn]

Qi Zhang [zhang@odu.edu]

For peer review only - http://bmjopen.bmj.com/site/about/guidelines.xhtml

ABSTRACT

Introduction Though evidence has revealed the beneficial effects of cognitive improvement interventions on breastfeeding, the effect of psychological interventions has rarely been studied. This study aims to test whether promoting a positive emotion intervention, “Three Good Things” intervention, during the last trimester of pregnancy can enhance early colostrum secretion and breastfeeding behaviors by modulating the hormones associated with lactation (prolactin and insulin-like growth factor Ⅰ). We will attempt to promote exclusive breastfeeding by using physiological behavioral measures.

Methods and analysis This study is designed as a randomized controlled trial conducted in the Women's Hospital School of Medicine at Zhejiang University and the Wuyi First People’s Hospital. The participants will be randomly divided into two groups using stratified random grouping: the intervention group will receive “Three Good Things” intervention while the control group will write about three things that come to mind first. These interventions will be continued from enrollment until the day of delivery. Maternal blood hormone levels will be tested approaching delivery and the following day after birth. Behavioral information about breastfeeding will be collected one week afterward.

Keywords Colostrum secretion; Positive psychology; Three good things; Breastfeeding; Randomized controlled trial.

Ethics and dissemination The study has been approved by the Ethics Committees of the Women's Hospital School of Medicine at Zhejiang University and the Wuyi First

For peer review only - http://bmjopen.bmj.com/site/about/guidelines.xhtml

People's Hospital. Results will be disseminated through peer-reviewed journals or international academic conferences.

Trial registration： Health management during pregnancy: research on breastfeeding promotion based on behavioural economics and positive psychological approaches, ChiCTR2000038849.

http://www.chictr.org.cn/usercenter.aspx

Strengths and limitations of this study

- A Randomized controlled trial of pregnant women will be performed to evaluate the effect of "Three Good Things" on their postpartum emotions, colostrum secretion and breastfeeding behaviors.
- "Three Good Things" is a typical psychological intervention used for promoting positive emotions, which has shown significant effect.
- "Three Good Things" is feasible to carry out by individuals and easy to disseminate in daily routines.
- We will promote breastfeeding-related behaviors in the postpartum hospitalization period in the setting of a Baby-Friendly hospital, in line with future trends in China.
- The results interpretations on breastfeeding behaviors are limited in postpartum period due to the limits of existing conditions.

Introduction

For peer review only - http://bmjopen.bmj.com/site/about/guidelines.xhtml

Breastfeeding is one of the most valid ways for promoting children's health and survival, which is advocated by the World Health Organization (WHO) and the United Nations Children's Fund (UNICEF)[1]. Breast milk, with its balanced nutrition, suitable temperature, and economic benefits, is considered the most ideal natural food for infants[2,3]. In addition, breast milk contains a diverse array of microbiota and myriad biologically-active components that contribute to the development of infants' immune systems. Thus, compared with formula-fed infants, breastfed children have fewer incidences of disease and lower mortality rates[4,5]. Breastfeeding also has economic benefits for individual families as well as at the national level by improving child development and reducing health costs[6]. As the WHO suggests, infants should be exclusively breastfed (given nothing else but breastmilk) for the first six months of life and be allowed to breastfeed to 2 years of age or longer[7]. In 2012, the 56th World Health Assembly set a goal for 2025 to increase exclusive breastfeeding rates in the first six months to at least 50 percent[8]. However, few countries in the world fully meet the recommended standards, according to a recent report by UNICEF and the WHO in collaboration with the Global Breastfeeding Collective, a new initiative to increase global breastfeeding rates[8]. The *Global Breastfeeding Scorecard,* which evaluated 194 nations, found that only about 40% of children are breastfed exclusively within the first six months of life, and only 23 countries exceed the standard of 60% on their exclusive breastfeeding rates[8].

To promote exclusive breastfeeding in the first 6 months postpartum, a large number of studies have explored the influencing factors and intervention measures. A

For peer review only

For peer review only - http://bmjopen.bmj.com/site/about/guidelines.xhtml

previous study indicated that a positive antenatal attitude toward breastfeeding was the strongest predictor and was associated with a 20-30% increase in breastfeeding initiation and maintenance at all time points[9]. Accordingly, the Breastfeeding Support Programme (BSP) was carried out to improve the attitudes and cognitions about breastfeeding among pregnant women. It was found to be an effective measure to delay the cessation of breastfeeding, increasing both its duration and its exclusivity[10].

In addition to cognitive factors, psychological status and emotions also play an important part in breastfeeding. Current evidence suggests that anxiety and depression are associated with lower exclusive breastfeeding behaviors[11–13]. Psychological distress resulted in low appetite, weight loss, and poor nutritional status, all of which made it difficult to breastfeed[14]. Emotional and traumatic stressors were found to have the greatest impact on breastfeeding outcomes[15]. Of clinical relevance, addressing prenatal and postpartum distress as a part of the implementation of breastfeeding practice interventions could improve breastfeeding rates[16]. Although intervention studies have been focusing on psychological distress, few studies have conducted positive psychology interventions to promote breastfeeding. Positive psychology interventions (PPIs) can enhance women's mental well-being during the perinatal period[17]. Thus, PPIs may be worth exploring as measures to promote breastfeeding behaviors[18]. The "Three Good Things", as a PPI instructs participants to write down three good things every night, was proposed by Seligman et al. in 2005. It helps to develop skills in regulating emotions[19], which can enhance the experience of positive emotions and alleviate the symptoms of depression[20]. This psychological intervention has been tested

For peer review only - http://bmjopen.bmj.com/site/about/guidelines.xhtml

in multiple studies and is replicable and easy to implement by individuals. Therefore, this study will use and evaluate the effect of "Three Good Things" as an intervention to enhance positive emotions and reduce negative emotions among pregnant women.

Breastfeeding behaviors are influenced by lactation capacity, which is reflected in hormone levels. Prolactin (PRL) is a protein hormone secreted by the anterior pituitary gland that can stimulate and maintain lactation. Insulin-like growth factor I (IGF-I) is a kind of polypeptide that can mediate the galactagogue of growth hormone. Previous studies have shown that emotions and the secretion of these two hormones are closely related. Positive emotions can promote the secretion of prolactin, while depression can lead to a prolactin secretion imbalance, and stress events and anxiety can also lead to the low secretion level of IGF-I[21–23]. This study aims to use “Three Good Things” intervention to promote positive emotions among pregnant women during mid- and late pregnancy, thus improving the secretion of lactation-related hormones and the initiation and continuation of breastfeeding behaviors.

For peer review only - http://bmjopen.bmj.com/site/about/guidelines.xhtml

Methods and analysis

Aims

The study is designed as a randomized controlled trial in China, to test whether the "Three Good Things" intervention during late pregnancy can enhance colostrum secretion and related breastfeeding behaviors and effects. We also aim to examine whether the association between prenatal emotions and postnatal breastfeeding behaviors are mediated by lactation-related hormones.

Hypotheses

Hypotheses 1a: "Three Good Things" interventions can promote the secretion of PRL and IGF-I by enhancing positive emotions and reducing negative emotions of pregnant women.

Hypotheses 1b: "Three Good Things" interventions can improve maternal breastfeeding willingness and postpartum in-hospital breastfeeding behaviors by enhancing positive emotions and reducing negative emotions.

Hypotheses 2a: Increased secretion of PRL and IGF-I will predict the promotion in breastfeeding behaviors and effects.

Hypotheses 2b: Positive emotions can indirectly promote breastfeeding behaviors during postpartum hospitalization through the secretion of PRL and IGF-I, while negative emotions have adverse effects.

Study Design

This is the protocol for a randomized controlled clinical trial. Between October 2020 and June 2021, a pilot study was carried out at the First People's Hospital of Wuyi

For peer review only - http://bmjopen.bmj.com/site/about/guidelines.xhtml

County in Zhejiang Province. During this time, a total of 96 pregnant women were recruited for the test of the designed survey questionnaire, including baseline survey and follow-up survey, and the intervention questionnaire. In January 2022, the formal trial was conducted in Women's Hospital School of Medicine at Zhejiang University. This study will have three phases: T0, baseline; T1, immediate postintervention; T2, follow-up (one week after delivery). The qualitative and quantitative data will be collected in three phases. T1 is a baseline survey collecting demographic information, data about existing positive and negative emotions, and their intention to breastfeed. T2 is the intervention process for the intervention and the control groups, with process variables of emotion and willingness collected every week. T3 is the pre- and post-natal hospitalization period when we will collect outcome indicators such as serum hormone levels, positive and negative emotions, intention to breastfeed, breastfeeding behavior, and other indicators. Figure 1 illustrates the overall study design.

Recruitment

The study will be conducted in the Women's Hospital School of Medicine at Zhejiang University. We will include primiparous pregnant women at 34 to 38 gestational weeks. The exclusion criteria are as follows:

- Women with no intention to give birth in a designated hospital.
- Women not giving birth for the first time.
- Women with a disease that prevents breastfeeding (cardiac insufficiency, infectious diseases, neurological or cognitive damage, et al.)
- Pregnant women with more than one fetus.

For peer review only - http://bmjopen.bmj.com/site/about/guidelines.xhtml

Sample size

Based on the results of the pilot study, statistical power (1-β) of 80%, and a two-tailed significance level (α) of 0.05, we calculated the targeted sample size with a two-sample t-test allowing unequal variance. Given the breastfeeding behavior score (LATCH) was 12.72 in the intervention group (standard deviation, SD = 2.33) and 12.06 in the control group (SD = 1.51), the theoretical sample size was shown as N1 = N2 = 141. Considering a dropout rate of 20%, the ultimate sample size needed was estimated at 353 individuals.

Randomization and blinding

Medical staff at the hospital's fetal heart monitoring studio will help recruit participants. We will use stratified random grouping to divide the pregnant women into two groups according to the level of support they say they receive (high, average, none) from family members in the oral interview. Baseline data will be collected before the randomization. Pregnant women received on the same day will be randomly assigned to the intervention group and the control group according to the level of support (high, average, none). KCX will generate the randomization list, and YX will carry out the randomization and give the participants information about which group they are allocated to. Our trial will be a single-blinded trial of participants.

Intervention

At fetal heart monitoring studio, we will ask the participants to add our Wechat account, and the subsequent communications and relevant questionnaire distribution will be carried out through Wechat chat software. In the T1 stage, participants will

For peer review only - http://bmjopen.bmj.com/site/about/guidelines.xhtml

193 complete a series of intervention questionnaires. Participants in the intervention group 194 will be asked to write down three good things that have happened during the day every 195 two days, with weekly specific themes. Participants in the control group will only have 196 to write about three things that come to mind first that are related to the same theme. 197 To improve the compliance and interest of the participants, we will set up different 198 instructions and themes for them weekly (Table 1). Meanwhile, if a participant forgets 199 to write, we will send out hints if they fail to complete the questionnaires. When the 200 participant is unwilling to take the initiative to fill in the questionnaire, we will ask for 201 reasons and send encouraging messages (e.g. about the benefits of participating in the 202 project). For ethical considerations of avoiding excessive disturbance, the participants 203 will be regarded as loss-to-follow-up, after three non-responses.

204 **Table 1.** Instructions for the Intervention and Control Groups.

Week	Topic	Instructions for the intervention group	Instructions for the control group
1	**My baby**	Please recall 3 good things that happened to you today related to you and your baby, and record them with the reasons why they happened. Here are some examples:<br>(1) My baby is moving today, and my baby is growing healthily.<br>(2) I saw a cute baby picture today, and my baby may be cuter.<br>(3) I bought baby's little clothes today, they are so small and cute.	Please record the first three baby-related events that come to your mind today, and record them and the reasons why they happened. Here are some examples:<br>(1) I suddenly thought that it is not easy to have a child.<br>(2) I had the maternity checkup today.
2	**Nature and me**	Please recall 3 good things that happened to you today related to nature, and record them with the reasons why they happened. Here are some examples:<br>(1) I heard a bird chirping on the road today, is it saying hello to me?<br>(2) The small flower we planted is blooming, we have taken good care of it.<br>(3) The sky is blue today, and there are traces	Please record the first three nature-related events that come to your mind today, and record them and the reasons why they happened. Here are some examples:<br>(1) It's raining today, and the air is wet.<br>(2) I watered the flowers at home.

For peer review only - http://bmjopen.bmj.com/site/about/guidelines.xhtml

		of airplanes flying over.	
**3**	**My family**	Please recall 3 good things that happened to you today related to your family, and record them with the reasons why they happened. Here are some examples:<br>(1) My husband is very considerate, and I feel very happy.<br>(2) My mother and mother-in-law specially cook duck for me, they always love me very much.<br>(3) All the people I love and I am expecting the baby together. My friends also told me just take it easy and not to be nervous.	Please record the first three family-related events that come to your mind today, and record them and the reasons why they happened. Here are some examples:<br>(1) My husband and I had dinner with our parents today.<br>(2) A good friend of mine got married today.
**4**	**My future**	Please recall 3 good things that happened to you today related to the future, and record them with the reasons why they happened. Here are some examples:<br>(1) Today, I saw other people's little kids, this time next year I can have my own baby!<br>(2) The weather forecast says the weather will be good, I'm looking forward to sunshine tomorrow.<br>(3) Tomorrow my favorite TV series will be updated.	Please record the first three future-related events that come to your mind today, and record them and the reasons why they happened. Here are some examples:<br>(1) We're going for a checkup tomorrow.<br>(2) Tomorrow my mother-in-law is coming to visit us.
**5**	**My life**	Please recall 3 good things that happened to you today related to your life, and record them with the reasons why they happened. Here are some examples:<br>(1) Today, I listened to my favorite songs, which gave me a lot of inspiration and memories.<br>(2) Today, I found a gentle sentence on my phone, which said, "I was born mediocre, and I was born proud. Let's be a happy ordinary person. "<br>(3) The emoticons my friend sent me today were so cute, I thought they were very interesting and funny.	Please record the first three life-related events that come to your mind today, and record them and the reasons why they happened. Here are some examples:<br>(1) I watch videos on the phone for an hour today.<br>(2) I watched a TV series with my husband this evening.

205 ***Evaluation and outcomes***

206 Primary outcomes will include exclusive breastfeeding and on-demand feeding during

207 postnatal hospitalization. Exclusive breastfeeding means that the baby is not given any

For peer review only - http://bmjopen.bmj.com/site/about/guidelines.xhtml

liquid or solid food other than breast milk. This indicator will be included in the postnatal questionnaire and also be double-checked in the hospital care records of formula. On-demand feeding means breastfeeding according to the baby's needs. Since the infant's early urination reflects breastfeeding, changing diapers 6-8 times a day in the early postpartum period is considered feeding on demand.

Secondary outcome indicators will mainly include other breastfeeding behaviors during postpartum hospitalization (first postnatal food for babies is breast milk; LATCH breastfeeding score), lactation-related hormone secretion (prolactin and IGF-I), and postpartum breastfeeding intention and postpartum emotion.

Data collection and follow-up

On the day of enrollment, pregnant women will be asked to complete an enrollment questionnaire providing information about general demographic characteristics and other factors influencing breastfeeding. Meanwhile, if the enrollment questionnaire finds prenatal depression among the participants, we will refer them to the department of psychology. Then the ecological instantaneous evaluation of both groups will be performed every week to determine the trend of change in intention and emotions until delivery. The measurement of emotion uses the Positive and Negative Affect Scale (PANAS) and 10 positive items for positive emotions proposed by Barbara L. Fredrickson for validation[24,25]. Postpartum questionnaires on breastfeeding behavior during postnatal hospitalization will be completed one week after delivery. Blood samples will be collected twice, after they are admitted to the hospital approaching delivery and after delivery at 7 to 8 a.m. (Vaginal Delivery: the morning after one night;

For peer review only - http://bmjopen.bmj.com/site/about/guidelines.xhtml

Cesarean section: the morning after two nights), and evaluated for prolactin and IGF-I to evaluate postpartum lactation ability. The blood draw will be conducted by nurses in the ward.

Upon recruitment, general demographic variables will be collected: education, occupation, number of permanent household members, total average annual household income, and expected date of birth. Other influencing factors will include mode of delivery, self-reported ease of breastfeeding, degree of sleep disturbance, self-reported importance of early exclusive breastfeeding, knowledge of breastfeeding, birth injury and nipple cracking pain scores, degree of hospital support, depression (measured by Edinburgh Postnatal Depression Scale, EPDS), anxiety (measured by Generalized Anxiety Disorder, GAD-7), breastfeeding intentions (measured by Infant Feeding Intentions Scale, IFI), breastfeeding behavior score (LATCH), resilience (Brief Resilience Scale, BRS), and potential number of traumatic events[26,27(p7),28–31]. Generally, Baby-friendly hospitals will take measures such as early sucking and skin-to-skin contact within 1hr after delivery, limitation of bottle used, room-in, etc[32]. We will also record and report routine measures taken in this Baby-Friendly hospital.

All questionnaires will be made through the questionnaire star website and distributed via WeChat. The corresponding data are stored in the questionnaire star account. Hormone test results will be recorded and saved manually. We will keep the personal information and data of each participant strictly confidential.

Data analysis

For peer review only - http://bmjopen.bmj.com/site/about/guidelines.xhtml

Descriptive analyses will be performed to show the baseline characteristics of the participants. To assess the intervention effects over time, MANOVA of repeated measuring will be taken for the change of positive and negative emotions, intention to breastfeed, and resilience trend across the weeks. To test the association among positive/negative emotions, lactation-related hormones, and breastfeeding behaviors, we will perform multiple linear regression or logistic regression in between and exploratory mediation analysis. To evaluate the effect of the intervention, we will take a two-sample T-test or Wilcoxon rank-sum test for quantitative data and a Chi-square test for qualitative data.

Data monitoring

The data monitoring committee is composed of members of the science and education Department of the hospital. As the administrative department of the hospital, the Ministry of Science and Education is in charge of scientific research, and reviews the integrity of scientific research to ensure the authenticity of data.

Patient and Public Involvement

The development of the research question and outcome measures isn't influenced by patients' priorities, experiences, and preferences. Participants and the public don't involve in the design, recruitment or conduct of the study. We will disseminate the results to study participants through subsequent publications. For this RCT, the burden of the intervention is assessed by the patients themselves through the questionnaire.

Discussion

Our study has several strengths. First, as distinct from current interventions focusing on

For peer review only - http://bmjopen.bmj.com/site/about/guidelines.xhtml

knowledge improvement, our study attempts to promote the secretion of lactation-related hormones by elevating positive emotions, then breastfeeding behaviors. Second, most previous interventions promoting breastfeeding were set in the postpartum period. Considering the importance of early breastfeeding experiences, we moved the intervention period to the late stages of pregnancy. Third, earlier mixed educational and supportive interventions are difficult to evaluate and replicate precisely. Our study will use the typical psychological measures, "Three Good Things", to boost positive emotions, which is feasible for individuals to carry out and can be easier to promote and popularize among pregnant women.

This study also has several limitations. First, due to the restrictions of the testing technology and the intricacies involved in distinguishing between endogenous and exogenous sources, we will not test for oxytocin in the serum, although this has been widely reported to be associated with lactation. However, oxytocin release is related to prolactin levels, which will be included in our test[33]. Second, we limit the outcome measurement of breastfeeding-related behaviors to the postpartum hospitalization period, without following a 6-month or longer period. Nevertheless, exclusive breastfeeding in the early postpartum period (1 hour and within a few days after delivery) has been positively correlated with the duration of breastfeeding after delivery.

In conclusion, this study will test the effect of "Three Good Things" intervention not only on the emotions of women during pregnancy, but also on the secretion of colostrum and breastfeeding behaviors as well. This study will also help us further identify the physiological mechanisms of the association between emotions and

For peer review only - http://bmjopen.bmj.com/site/about/guidelines.xhtml

breastfeeding behaviors.

List of abbreviations

WHO	(World Health Organization)
UNICEF	(United Nations Children's Fund)
IGF-I	(Insulin-like growth factor Ⅰ)
PRL	(Prolactin)
PANAS	(Positive and Negative Affect Scale)
EPDS	(Edinburgh Postnatal Depression Scale)
GAD-7	(Generalized Anxiety Disorder-7)
IFI	(Infant Feeding Intentions Scale)
BRS	(Brief Resilience Scale)

Ethics and dissemination

The study has been approved by the Ethics Committees of the Women's Hospital School of Medicine at Zhejiang University (20190088) and the Wuyi First People's Hospital. The study will be conducted by the Declaration of Helsinki ethical principles for research involving human subjects. Results will be disseminated through peer-reviewed journals or international academic conferences. Written consent will be obtained from all participants.

Footnotes

Availability of data and materials

Not applicable.

Authors' contributions

KCX, YX, XJH and YY contributed to data acquisition and analysis. KCX and YX were in charge of the writing. SQL and SLW participated in the implementation of the

For peer review only - http://bmjopen.bmj.com/site/about/guidelines.xhtml

pilot trial. QY and QZ contributed to study design and manuscript revision. All authors approved the final version of the manuscript.

Funding statement

This study is supported by the National Natural Science Foundation of China (71974170), Medical Scientific Research Foundation of Zhejiang Province of China (2021455676). The funders have no influences on study methods, analysis, interpretation, or publication of results.

Competing interests statement

The authors declare no conflict of interest. The funders have no influences on study methods, analysis, interpretation, or publication of results.

Patient consent for publication

Not required.

Acknowledgements

We appreciate the support of the clinical staff of Zhejiang University School of Medicine and Wuyi First People's Hospital, including Xinyi Zhou, Suqing Liu, Xiaohong Qin, Qin Geng, and Di Gu.

Figure 1. Flow chart of the breastfeeding intervention project.The participants will be randomly divided into intervention group and control group. In phase T0, participants will complete the first questionnaire, including breastfeed intention, emotional scale, and writing practice persistence. In the T1 stage, the participants will complete a series of intervention questionnaires, including breastfeed intention, emotional scale, writing practice persistence. First lactation hormone test will be performed before delivery. The second lactation hormone test will be performed after delivery. Finally, a postpartum questionnaire will be performed about the outcome of lactation behavior.

For peer review only - http://bmjopen.bmj.com/site/about/guidelines.xhtml

References

1. Infant and young child feeding. Accessed December 27, 2021. https://www.who.int/en/news-room/fact-sheets/detail/infant-and-young-child-feeding
2. Andreas NJ, Kampmann B, Mehring Le-Doare K. Human breast milk: A review on its composition and bioactivity. *Early Hum Dev*. 2015;91(11):629-635. doi:10.1016/j.earlhumdev.2015.08.013
3. Johnson TJ, Patel AL, Bigger HR, Engstrom JL, Meier PP. Economic benefits and costs of human milk feedings: a strategy to reduce the risk of prematurity-related morbidities in very-low-birth-weight infants. *Adv Nutr Bethesda Md*. 2014;5(2):207-212. doi:10.3945/an.113.004788
4. Le Doare K, Holder B, Bassett A, Pannaraj PS. Mother's Milk: A Purposeful Contribution to the Development of the Infant Microbiota and Immunity. *Front Immunol*. 2018;9:361. doi:10.3389/fimmu.2018.00361
5. Lyons KE, Ryan CA, Dempsey EM, Ross RP, Stanton C. Breast Milk, a Source of Beneficial Microbes and Associated Benefits for Infant Health. *Nutrients*. 2020;12(4):E1039. doi:10.3390/nu12041039
6. The Lancet Breastfeeding Series papers . 2016.
7. Global strategy for infant and young child feeding,WHO.
8. Babies and mothers worldwide failed by lack of investment in breastfeeding. Accessed December 27, 2021. https://www.who.int/news/item/01-08-2017-babies-and-mothers-worldwide-failed-by-lack-of-investment-in-breastfeeding
9. Amiel Castro RT, Glover V, Ehlert U, O'Connor TG. Antenatal psychological and socioeconomic predictors of breastfeeding in a large community sample. *Early Hum Dev*. 2017;110:50-56. doi:10.1016/j.earlhumdev.2017.04.010
10. van Dellen SA, Wisse B, Mobach MP, Dijkstra A. The effect of a breastfeeding support programme on breastfeeding duration and exclusivity: a quasi-experiment. *BMC Public Health*. 2019;19(1):993. doi:10.1186/s12889-019-7331-y
11. Ystrom E. Breastfeeding cessation and symptoms of anxiety and depression: a longitudinal cohort study. *BMC Pregnancy Childbirth*. 2012;12:36. doi:10.1186/1471-2393-12-36
12. Seimyr L, Edhborg M, Lundh W, Sjögren B. In the shadow of maternal depressed mood: experiences of parenthood during the first year after childbirth. *J Psychosom Obstet Gynaecol*. 2004;25(1):23-34. doi:10.1080/01674820410001737414
13. Kehler HL, Chaput KH, Tough SC. Risk factors for cessation of breastfeeding prior to six months postpartum among a community sample of women in Calgary, Alberta. *Can J Public Health Rev Can Sante Publique*. 2009;100(5):376-380.
14. Emerson JA, Tol W, Caulfield LE, Doocy S. Maternal Psychological Distress and Perceived Impact on Child Feeding Practices in South Kivu, DR Congo. *Food Nutr Bull*. 2017;38(3):319-337. doi:10.1177/0379572117714385
15. Kitsantas P, Gaffney KF, Nirmalraj L, Sari M. The influence of maternal life stressors on breastfeeding outcomes: a US population-based study. *J Matern-Fetal Neonatal Med Off J Eur Assoc Perinat Med Fed Asia Ocean Perinat Soc Int Soc Perinat Obstet*. 2019;32(11):1869-1873. doi:10.1080/14767058.2017.1421929
16. Ritchie-Ewing G, Mitchell AM, Christian LM. Associations of Maternal Beliefs

For peer review only - http://bmjopen.bmj.com/site/about/guidelines.xhtml

and Distress in Pregnancy and Postpartum With Breastfeeding Initiation and Early Cessation. *J Hum Lact Off J Int Lact Consult Assoc*. 2019;35(1):49-58. doi:10.1177/0890334418767832

17. Corno G, Espinoza M, Maria Baños R. A narrative review of positive psychology interventions for women during the perinatal period. *J Obstet Gynaecol J Inst Obstet Gynaecol*. 2019;39(7):889-895. doi:10.1080/01443615.2019.1581735
18. Shariat M, Abedinia N. The Effect of Psychological Intervention on Mother-Infant Bonding and Breastfeeding. *Iran J Neonatol*. 2017;8(1):7-15. doi:10.22038/ijn.2017.16673.1191
19. Seligman MEP, Steen TA, Park N, Peterson C. Positive psychology progress: empirical validation of interventions. *Am Psychol*. 2005;60(5):410-421. doi:10.1037/0003-066X.60.5.410
20. Beanlands H, McCay E, Fredericks S, et al. Decreasing stress and supporting emotional well-being among senior nursing students: A pilot test of an evidence-based intervention. *Nurse Educ Today*. 2019;76:222-227. doi:10.1016/j.nedt.2019.02.009
21. Turner RA, Altemus M, Yip DN, et al. Effects of emotion on oxytocin, prolactin, and ACTH in women. *Stress Amst Neth*. 2002;5(4):269-276. doi:10.1080/102538902100037586-1
22. Nicholas L, Dawkins K, Golden RN. Psychoneuroendocrinology of depression. Prolactin. *Psychiatr Clin North Am*. 1998;21(2):341-358. doi:10.1016/s0193-953x(05)70009-5
23. Field T. Prenatal anxiety effects: A review. *Infant Behav Dev*. 2017;49:120-128. doi:10.1016/j.infbeh.2017.08.008
24. Watson D, Clark LA, Tellegen A. Development and validation of brief measures of positive and negative affect: the PANAS scales. *J Pers Soc Psychol*. 1988;54(6):1063-1070. doi:10.1037//0022-3514.54.6.1063
25. Fredrickson BL. Positive Emotions Broaden and Build. In: Devine P, Plant A, eds. *Advances in Experimental Social Psychology, Vol 47*. Vol 47. Elsevier Academic Press Inc; 2013:1-53. doi:10.1016/B978-0-12-407236-7.00001-2
26. Cox JL, Holden JM, Sagovsky R. Detection of postnatal depression. Development of the 10-item Edinburgh Postnatal Depression Scale. *Br J Psychiatry J Ment Sci*. 1987;150:782-786. doi:10.1192/bjp.150.6.782
27. Spitzer RL, Kroenke K, Williams JBW, Löwe B. A brief measure for assessing generalized anxiety disorder: the GAD-7. *Arch Intern Med*. 2006;166(10):1092-1097. doi:10.1001/archinte.166.10.1092
28. Nommsen-Rivers LA, Dewey KG. Development and validation of the infant feeding intentions scale. *Matern Child Health J*. 2009;13(3):334-342. doi:10.1007/s10995-008-0356-y
29. Jensen D, Wallace S, Kelsay P. LATCH: a breastfeeding charting system and documentation tool. *J Obstet Gynecol Neonatal Nurs JOGNN*. 1994;23(1):27-32. doi:10.1111/j.1552-6909.1994.tb01847.x
30. Smith BW, Dalen J, Wiggins K, Tooley E, Christopher P, Bernard J. The brief resilience scale: assessing the ability to bounce back. *Int J Behav Med*. 2008;15(3):194-200. doi:10.1080/10705500802222972

For peer review only

For peer review only - http://bmjopen.bmj.com/site/about/guidelines.xhtml

31. Peterson C, Park N, Pole N, D'Andrea W, Seligman MEP. Strengths of character and posttraumatic growth. *J Trauma Stress*. 2008;21(2):214-217. doi:10.1002/jts.20332
32. Li L, Song H, Zhang Y, et al. Breastfeeding Supportive Services in Baby-Friendly Hospitals Positively Influenced Exclusive Breastfeeding Practice at Hospitalization Discharge and Six Months Postpartum. *Int J Environ Res Public Health*. 2021;18(21):11430. doi:10.3390/ijerph182111430
33. Uvnäs Moberg K, Ekström-Bergström A, Buckley S, et al. Maternal plasma levels of oxytocin during breastfeeding-A systematic review. *PloS One*. 2020;15(8):e0235806. doi:10.1371/journal.pone.0235806

For peer review only - http://bmjopen.bmj.com/site/about/guidelines.xhtml

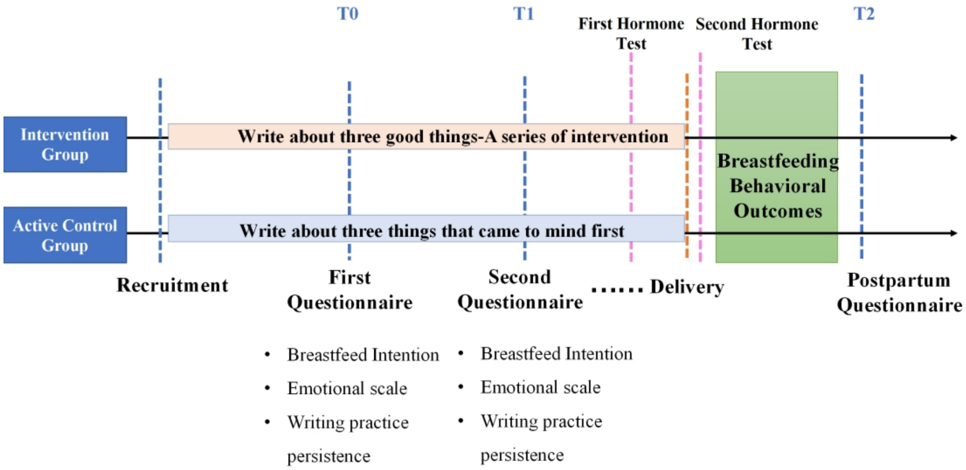

338x190mm (192 x 192 DPI)

STANDARD PROTOCOL ITEMS: RECOMMENDATIONS FOR INTERVENTIONAL TRIALS

SPIRIT 2013 Checklist: Recommended items to address in a clinical trial protocol and related documents*

Section/item	ItemNo	Description	
**Administrative information**			
Title	1	Descriptive title identifying the study design, population, interventions, and, if applicable, trial acronym	Title, Page 1
Trial registration	2a	Trial identifier and registry name. If not yet registered, name of intended registry	Trial registration, Page 2
	2b	All items from the World Health Organization Trial Registration Data Set	Trial registration, Page 2
Protocol version	3	Date and version identifier	NA
Funding	4	Sources and types of financial, material, and other support	Funding, Page14
Roles and responsibilities	5a	Names, affiliations, and roles of protocol contributors	Authors' contributions, Page14
	5b	Name and contact information for the trial sponsor	Correspondence, Page1
	5c	Role of study sponsor and funders, if any, in study design; collection, management, analysis, and interpretation of data; writing of the report; and the decision to submit the report for publication, including whether they will have ultimate authority over any of these activities	Competing interests, Page14
	5d	Composition, roles, and responsibilities of the coordinating centre, steering committee, endpoint adjudication committee, data management team, and other individuals or groups overseeing the trial, if applicable (see Item 21a for data monitoring committee)	NA
**Introduction**			
Background and rationale	6a	Description of research question and justification for undertaking the trial, including summary of relevant studies (published and unpublished) examining benefits and harms for each intervention	Background, paragraph 2-3
	6b	Explanation for choice of comparators	NA

For peer review only - http://bmjopen.bmj.com/site/about/guidelines.xhtml

Objectives	7	Specific objectives or hypotheses	Background, paragraph 4
Trial design	8	Description of trial design including type of trial (eg, parallel group, crossover, factorial, single group), allocation ratio, and framework (eg, superiority, equivalence, noninferiority, exploratory)	Methods/Design, paragraph 4

Methods: Participants, interventions, and outcomes

Study setting	9	Description of study settings (eg, community clinic, academic hospital) and list of countries where data will be collected. Reference to where list of study sites can be obtained	Study Design, Page 7-8
Eligibility criteria	10	Inclusion and exclusion criteria for participants. If applicable, eligibility criteria for study centres and individuals who will perform the interventions (eg, surgeons, psychotherapists)	Recruitment, Page 8
Interventions	11a	Interventions for each group with sufficient detail to allow replication, including how and when they will be administered	Intervention, Page 9-10
	11b	Criteria for discontinuing or modifying allocated interventions for a given trial participant (eg, drug dose change in response to harms, participant request, or improving/worsening disease)	NA
	11c	Strategies to improve adherence to intervention protocols, and any procedures for monitoring adherence (eg, drug tablet return, laboratory tests)	Intervention, Page 9-10
	11d	Relevant concomitant care and interventions that are permitted or prohibited during the trial	NA
Outcomes	12	Primary, secondary, and other outcomes, including the specific measurement variable (eg, systolic blood pressure), analysis metric (eg, change from baseline, final value, time to event), method of aggregation (eg, median, proportion), and time point for each outcome. Explanation of the clinical relevance of chosen efficacy and harm outcomes is strongly recommended	Evaluation and outcomes, Page 10
Participant timeline	13	Time schedule of enrolment, interventions (including any run-ins and washouts), assessments, and visits for participants. A schematic diagram is highly recommended (see Figure)	Data collection and follow-up, Page 10-11

For peer review only - http://bmjopen.bmj.com/site/about/guidelines.xhtml

Sample size	14	Estimated number of participants needed to achieve study objectives and how it was determined, including clinical and statistical assumptions supporting any sample size calculations	Sample size, Page 9
Recruitment	15	Strategies for achieving adequate participant enrolment to reach target sample size	Randomization and blinding, Page 9

Methods: Assignment of interventions (for controlled trials)

Allocation:

Sequence generation	16a	Method of generating the allocation sequence (eg, computer-generated random numbers), and list of any factors for stratification. To reduce predictability of a random sequence, details of any planned restriction (eg, blocking) should be provided in a separate document that is unavailable to those who enrol participants or assign interventions	Randomization and blinding, Page 9
Allocation concealme nt mechanism	16b	Mechanism of implementing the allocation sequence (eg, central telephone; sequentially numbered, opaque, sealed envelopes), describing any steps to conceal the sequence until interventions are assigned	Randomization and blinding, Page 9
Implementa tion	16c	Who will generate the allocation sequence, who will enrol participants, and who will assign participants to interventions	Randomization and blinding, Page 9
Blinding (masking)	17a	Who will be blinded after assignment to interventions (eg, trial participants, care providers, outcome assessors, data analysts), and how	Randomization and blinding, Page 9
	17b	If blinded, circumstances under which unblinding is permissible, and procedure for revealing a participant's allocated intervention during the trial	NA

Methods: Data collection, management, and analysis

Data collection methods	18a	Plans for assessment and collection of outcome, baseline, and other trial data, including any related processes to promote data quality (eg, duplicate measurements, training of assessors) and a description of study instruments (eg, questionnaires, laboratory tests) along with their reliability and validity, if known. Reference to where data collection forms can be found, if not in the protocol	Data collection and follow-up, Page 10-11
	18b	Plans to promote participant retention and complete follow-up, including list of any outcome data to be collected for participants who discontinue or deviate from intervention protocols	Intervention, Page 9-10

Data management	19	Plans for data entry, coding, security, and storage, including any related processes to promote data quality (eg, double data entry; range checks for data values). Reference to where details of data management procedures can be found, if not in the protocol	Data collection and follow-up, Page 10-11
Statistical methods	20a	Statistical methods for analysing primary and secondary outcomes. Reference to where other details of the statistical analysis plan can be found, if not in the protocol	Data analysis, Page 11-12
	20b	Methods for any additional analyses (eg, subgroup and adjusted analyses)	NA
	20c	Definition of analysis population relating to protocol non-adherence (eg, as randomised analysis), and any statistical methods to handle missing data (eg, multiple imputation)	NA
**Methods: Monitoring**			
Data monitoring	21a	Composition of data monitoring committee (DMC); summary of its role and reporting structure; statement of whether it is independent from the sponsor and competing interests; and reference to where further details about its charter can be found, if not in the protocol. Alternatively, an explanation of why a DMC is not needed	Data monitoring, Page 12
	21b	Description of any interim analyses and stopping guidelines, including who will have access to these interim results and make the final decision to terminate the trial	NA
Harms	22	Plans for collecting, assessing, reporting, and managing solicited and spontaneously reported adverse events and other unintended effects of trial interventions or trial conduct	NA
Auditing	23	Frequency and procedures for auditing trial conduct, if any, and whether the process will be independent from investigators and the sponsor	NA
**Ethics and dissemination**			
Research ethics approval	24	Plans for seeking research ethics committee/institutional review board (REC/IRB) approval	Ethics approval and consent to participate, Page 14
Protocol amendments	25	Plans for communicating important protocol modifications (eg, changes to eligibility criteria, outcomes, analyses) to relevant parties (eg, investigators, REC/IRBs, trial participants, trial registries, journals, regulators)	NA

For peer review only - http://bmjopen.bmj.com/site/about/guidelines.xhtml

Consent or assent	26a	Who will obtain informed consent or assent from potential trial participants or authorised surrogates, and how (see Item 32)	Ethics approval and consent to participate, Page 14
	26b	Additional consent provisions for collection and use of participant data and biological specimens in ancillary studies, if applicable	NA
Confidentiality	27	How personal information about potential and enrolled participants will be collected, shared, and maintained in order to protect confidentiality before, during, and after the trial	Intervention, Data collection and follow-up, Page 9-11
Declaration of interests	28	Financial and other competing interests for principal investigators for the overall trial and each study site	Competing interests, Page 14
Access to data	29	Statement of who will have access to the final trial dataset, and disclosure of contractual agreements that limit such access for investigators	NA
Ancillary and post-trial care	30	Provisions, if any, for ancillary and post-trial care, and for compensation to those who suffer harm from trial participation	NA
Dissemination policy	31a	Plans for investigators and sponsor to communicate trial results to participants, healthcare professionals, the public, and other relevant groups (eg, via publication, reporting in results databases, or other data sharing arrangements), including any publication restrictions	NA
	31b	Authorship eligibility guidelines and any intended use of professional writers	NA
	31c	Plans, if any, for granting public access to the full protocol, participant-level dataset, and statistical code	NA
**Appendices**			
Informed consent materials	32	Model consent form and other related documentation given to participants and authorised surrogates	Informed consent materials
Biological specimens	33	Plans for collection, laboratory evaluation, and storage of biological specimens for genetic or molecular analysis in the current trial and for future use in ancillary studies, if applicable	NA

*It is strongly recommended that this checklist be read in conjunction with the SPIRIT 2013 Explanation & Elaboration for important clarification on the items. Amendments to the protocol should be tracked and dated. The SPIRIT checklist is copyrighted by the SPIRIT

For peer review only - http://bmjopen.bmj.com/site/about/guidelines.xhtml

Group under the Creative Commons "Attribution-NonCommercial-NoDerivs 3.0 Unported" license.

For peer review only

For peer review only - http://bmjopen.bmj.com/site/about/guidelines.xhtml

BMJ Open

The effect of positive emotion intervention during late pregnancy on improving colostrum secretion: a randomized control trial protocol

Journal:	*BMJ Open*
Manuscript ID	bmjopen-2022-066601.R2
Article Type:	Protocol
Date Submitted by the Author:	04-Jun-2023
Complete List of Authors:	Xu, Kechen; Zhejiang University School of Medicine, School of Public Health, ; Wuyi First People’s Hospital, Clinical laboratory center<br>Xie, Ying ; Chinese Academy of Medical Sciences & Peking Union Medical College, School of Health Policy and Management<br>Han, Xiujun; Women's Hospital School of Medicine Zhejiang University<br>Yu, Ying; Women's Hospital School of Medicine Zhejiang University<br>Liu, Suqing; Wuyi First People’s Hospital<br>Wu, Suliu; Wuyi First People’s Hospital<br>Yang, Qian; Zhejiang University School of Public Health, Center for Health Policy Studies; The Fourth Affiliated Hospital Zhejiang University School of Medicine, the department of Geriatrics<br>Zhang, Qi; Old Dominion University, Community & Environmental Health
**Primary Subject Heading**:	Obstetrics and gynaecology
Secondary Subject Heading:	Public health, Mental health
Keywords:	PUBLIC HEALTH, PSYCHIATRY, OBSTETRICS

SCHOLARONE™
Manuscripts

The effect of positive emotion intervention during late pregnancy on improving colostrum secretion: a randomized control trial protocol

Kechen Xu[1,2,a], Ying Xie[3,a], Xiujun Han[4,a], Ying Yu[4,a], Suqing Liu[2], Suliu Wu[2], Qian Yang[1,*], Qi Zhang[5]

1. School of Public Health and the Department of Geriatrics, the Fourth Affiliated Hospital, Zhejiang University School of Medicine, Zhejiang University, Zhejiang University, Hangzhou, 310058, China, CN

2. Wuyi First People's Hospital, Wuyi, Jinhua 321200, Zhejiang, China

3. School of Health Policy and Management, Chinese Academy of Medical Sciences and Peking Union Medical College, Beijing, China

4. Women's Hospital School of Medicine Zhejiang University, Hangzhou, Zhejiang, China

5. Community & Environmental Health, Old Dominion University, Norfolk, Virginia, USA

a. These authors contributed equally to this article.

* Correspondence

Qian Yang [chianyoung@zju.edu.cn]

For peer review only - http://bmjopen.bmj.com/site/about/guidelines.xhtml

ABSTRACT

Introduction Though evidence has revealed the beneficial effects of cognitive improvement interventions on breastfeeding, the effect of psychological interventions has rarely been studied. This study aims to test whether promoting a positive emotion intervention, “Three Good Things” intervention, during the last trimester of pregnancy can enhance early colostrum secretion and breastfeeding behaviors by modulating the hormones associated with lactation (prolactin and insulin-like growth factor Ⅰ). We will attempt to promote exclusive breastfeeding by using physiological behavioral measures.

Methods and analysis This study is designed as a randomized controlled trial conducted in the Women's Hospital School of Medicine at Zhejiang University and the Wuyi First People’s Hospital. The participants will be randomly divided into two groups using stratified random grouping: the intervention group will receive “Three Good Things” intervention while the control group will write about three things that come to mind first. These interventions will be continued from enrollment until the day of delivery. Maternal blood hormone levels will be tested approaching delivery and the following day after birth. Behavioral information about breastfeeding will be collected one week afterward.

Ethics and dissemination The study has been approved by the Ethics Committees of the Women's Hospital School of Medicine at Zhejiang University and the Wuyi First People’s Hospital. Results will be disseminated through peer-reviewed journals or international academic conferences.

For peer review only - http://bmjopen.bmj.com/site/about/guidelines.xhtml

Trial registration Health management during pregnancy: research on breastfeeding promotion based on behavioural economics and positive psychological approaches, ChiCTR2000038849.

http://www.chictr.org.cn/usercenter.aspx

Strengths and limitations of this study

- A Randomized controlled trial of pregnant women will be performed to evaluate the effect of "Three Good Things" on their postpartum emotions, colostrum secretion and breastfeeding behaviors.
- "Three Good Things" is a typical psychological intervention used for promoting positive emotions, which has shown significant effects.
- "Three Good Things" is feasible to carry out by individuals and easy to disseminate in daily routines.
- We will promote breastfeeding-related behaviors in the postpartum hospitalization period in the setting of a Baby-Friendly hospital, in line with future trends in China.
- The results interpretations on breastfeeding behaviors are limited in postpartum period due to the limits of existing conditions.

Keywords Colostrum secretion; Positive psychology; Three good things; Breastfeeding; Randomized controlled trial.

Introduction

For peer review only - http://bmjopen.bmj.com/site/about/guidelines.xhtml

Breastfeeding is one of the most valid ways for promoting children's health and survival, which is advocated by the World Health Organization (WHO) and the United Nations Children's Fund (UNICEF)[1]. Breast milk, with its balanced nutrition, suitable temperature, and economic benefits, is considered the most ideal natural food for infants[2,3]. In addition, breast milk contains a diverse array of microbiota and myriad biologically-active components that contribute to the development of infants' immune systems. Thus, compared with formula-fed infants, breastfed children have fewer incidences of disease and lower mortality rates[4,5]. Breastfeeding also has economic benefits for individual families as well as at the national level by improving child development and reducing health costs[6]. As the WHO suggests, infants should be exclusively breastfed (given nothing else but breastmilk) for the first six months of life and be allowed to breastfeed to 2 years of age or longer[7]. In 2012, the 56th World Health Assembly set a goal for 2025 to increase exclusive breastfeeding rates in the first six months to at least 50 percent[8]. However, few countries in the world fully meet the recommended standards, according to a recent report by UNICEF and the WHO in collaboration with the Global Breastfeeding Collective, a new initiative to increase global breastfeeding rates[8]. The *Global Breastfeeding Scorecard,* which evaluated 194 nations, found that only about 40% of children are breastfed exclusively within the first six months of life, and only 23 countries exceed the standard of 60% on their exclusive breastfeeding rates[8].

To promote exclusive breastfeeding in the first 6 months postpartum, a large number of studies have explored the influencing factors and intervention measures. A

For peer review only - http://bmjopen.bmj.com/site/about/guidelines.xhtml

previous study indicated that a positive antenatal attitude toward breastfeeding was the strongest predictor and was associated with a 20-30% increase in breastfeeding initiation and maintenance at all time points[9]. Accordingly, the Breastfeeding Support Programme (BSP) was carried out to improve the attitudes and cognitions about breastfeeding among pregnant women. It was found to be an effective measure to delay the cessation of breastfeeding, increasing both its duration and its exclusivity[10].

In addition to cognitive factors, psychological status and emotions also play an important part in breastfeeding. Current evidence suggests that anxiety and depression are associated with lower exclusive breastfeeding behaviors[11–13]. Psychological distress resulted in low appetite, weight loss, and poor nutritional status, all of which made it difficult to breastfeed[14]. Emotional and traumatic stressors were found to have the greatest impact on breastfeeding outcomes[15]. Of clinical relevance, addressing prenatal and postpartum distress as a part of the implementation of breastfeeding practice interventions could improve breastfeeding rates[16]. Although intervention studies have been focusing on psychological distress, few studies have conducted positive psychology interventions to promote breastfeeding. Positive psychology interventions (PPIs) can enhance women's mental well-being during the perinatal period[17]. Thus, PPIs may be worth exploring as measures to promote breastfeeding behaviors[18]. The "Three Good Things", as a PPI instructs participants to write down three good things every night, was proposed by Seligman et al. in 2005. It helps to develop skills in regulating emotions[19], which can enhance the experience of positive emotions and alleviate the symptoms of depression[20]. This psychological intervention has been tested

For peer review only - http://bmjopen.bmj.com/site/about/guidelines.xhtml

in multiple studies and is replicable and easy to implement by individuals. Therefore, this study will use and evaluate the effect of "Three Good Things" as an intervention to enhance positive emotions and reduce negative emotions among pregnant women.

Breastfeeding behaviors are influenced by lactation capacity, which is reflected in hormone levels. Prolactin (PRL) is a protein hormone secreted by the anterior pituitary gland that can stimulate and maintain lactation. Insulin-like growth factor I (IGF-I) is a kind of polypeptide that can mediate the galactagogue of growth hormone. Previous studies have shown that emotions and the secretion of these two hormones are closely related. Positive emotions can promote the secretion of prolactin, while depression can lead to a prolactin secretion imbalance, and stress events and anxiety can also lead to a low secretion level of IGF-I[21–23]. This study aims to use “Three Good Things” intervention to promote positive emotions among pregnant women during mid- and late pregnancy, thus improving the secretion of lactation-related hormones and the initiation and continuation of breastfeeding behaviors.

For peer review only

For peer review only - http://bmjopen.bmj.com/site/about/guidelines.xhtml

Methods and analysis

Aims

The study is designed as a randomized controlled trial in China, to test whether the "Three Good Things" intervention during late pregnancy can enhance colostrum secretion and related breastfeeding behaviors and effects. We also aim to examine whether the association between prenatal emotions and postnatal breastfeeding behaviors are mediated by lactation-related hormones.

Hypotheses

Hypotheses 1a: "Three Good Things" interventions can promote the secretion of PRL and IGF-I by enhancing positive emotions and reducing negative emotions in pregnant women.

Hypotheses 1b: "Three Good Things" interventions can improve maternal breastfeeding willingness and postpartum in-hospital breastfeeding behaviors by enhancing positive emotions and reducing negative emotions.

Hypotheses 2a: Increased secretion of PRL and IGF-I will predict the promotion of breastfeeding behaviors and effects.

Hypotheses 2b: Positive emotions can indirectly promote breastfeeding behaviors during postpartum hospitalization through the secretion of PRL and IGF-I, while negative emotions have adverse effects.

Study Design

This is the protocol for a randomized controlled clinical trial. Between October 2020 and June 2021, a pilot study was carried out at the First People's Hospital of Wuyi

For peer review only - http://bmjopen.bmj.com/site/about/guidelines.xhtml

County in Zhejiang Province. During this time, a total of 96 pregnant women were recruited for the test of the designed survey questionnaire, including the baseline survey and follow-up survey, and intervention questionnaire. In January 2022, the formal trial was conducted at Women's Hospital School of Medicine at Zhejiang University. This study will have three phases: T0, baseline; T1, immediate postintervention; T2, follow-up (one week after delivery). The qualitative and quantitative data will be collected in three phases. T1 is a baseline survey collecting demographic information, data about existing positive and negative emotions, and their intention to breastfeed. T2 is the intervention process for the intervention and the control groups, with process variables of emotion and willingness collected every week. T3 is the pre- and post-natal hospitalization period when we will collect outcome indicators such as serum hormone levels, positive and negative emotions, intention to breastfeed, breastfeeding behavior, and other indicators. Figure 1 illustrates the overall study design.

Recruitment

The study will be conducted in the Women's Hospital School of Medicine at Zhejiang University. We will include primiparous pregnant women at 34 to 38 gestational weeks. The exclusion criteria are as follows:

- Women with no intention to give birth in a designated hospital.
- Women not giving birth for the first time.
- Women with a disease that prevents breastfeeding (cardiac insufficiency, infectious diseases, neurological or cognitive damage, et al.)
- Pregnant women with more than one fetus.

For peer review only - http://bmjopen.bmj.com/site/about/guidelines.xhtml

Sample size

Based on the results of the pilot study, statistical power (1-β) of 80%, and a two-tailed significance level (α) of 0.05, we calculated the targeted sample size with a two-sample t-test allowing unequal variance. Given the breastfeeding behavior score (LATCH) was 12.72 in the intervention group (standard deviation, SD = 2.33) and 12.06 in the control group (SD = 1.51), the theoretical sample size was shown as N1 = N2 = 141. Considering a dropout rate of 20%, the ultimate sample size needed was estimated at 353 individuals.

Randomization and blinding

Medical staff at the hospital's fetal heart monitoring studio will help recruit participants. We will use stratified random grouping to divide the pregnant women into two groups according to the level of support they say they receive (high, average, none) from family members in the oral interview. Baseline data will be collected before the randomization. Pregnant women received on the same day will be randomly assigned to the intervention group and the control group according to the level of support (high, average, none). KCX will generate the randomization list, and YX will carry out the randomization and give the participants information about which group they are allocated to. Our trial will be a single-blinded trial of participants.

Intervention

At the fetal heart monitoring studio, we will ask the participants to add our Wechat account, and the subsequent communications and relevant questionnaire distribution will be carried out through Wechat chat software. In the T1 stage, participants will

For peer review only

For peer review only - http://bmjopen.bmj.com/site/about/guidelines.xhtml

complete a series of intervention questionnaires. Participants in the intervention group will be asked to write down three good things that have happened during the day every two days, with weekly specific themes. Participants in the control group will only have to write about three things that come to mind first that are related to the same theme. To improve the compliance and interest of the participants, we will set up different instructions and themes for them weekly (Table 1). Meanwhile, if a participant forgets to write, we will send out hints if they fail to complete the questionnaires. When the participant is unwilling to take the initiative to fill in the questionnaire, we will ask for reasons and send encouraging messages (e.g. about the benefits of participating in the project). For ethical considerations of avoiding excessive disturbance, the participants will be regarded as loss-to-follow-up, after three non-responses.

Table 1. Instructions for the Intervention and Control Groups.

Week	Topic	Instructions for the intervention group	Instructions for the control group
1	**My baby**	Please recall 3 good things that happened to you today related to you and your baby, and record them with the reasons why they happened. Here are some examples:<br>(1) My baby is moving today, and my baby is growing healthily.<br>(2) I saw a cute baby picture today, and my baby may be cuter.<br>(3) I bought baby's little clothes today, they are so small and cute.	Please record the first three baby-related events that come to your mind today, and record them and the reasons why they happened. Here are some examples:<br>(1) I suddenly thought that it is not easy to have a child.<br>(2) I had the maternity checkup today.
2	**Nature and me**	Please recall 3 good things that happened to you today related to nature, and record them with the reasons why they happened. Here are some examples:<br>(1) I heard a bird chirping on the road today, is it saying hello to me?<br>(2) The small flower we planted is blooming, we have taken good care of it.<br>(3) The sky is blue today, and there are traces	Please record the first three nature-related events that come to your mind today, and record them and the reasons why they happened. Here are some examples:<br>(1) It's raining today, and the air is wet.<br>(2) I watered the flowers at home.

For peer review only - http://bmjopen.bmj.com/site/about/guidelines.xhtml

		of airplanes flying over.	
**3**	**My family**	Please recall 3 good things that happened to you today related to your family, and record them with the reasons why they happened. Here are some examples:<br>(1) My husband is very considerate, and I feel very happy.<br>(2) My mother and mother-in-law specially cook duck for me, they always love me very much.<br>(3) All the people I love and I am expecting the baby together. My friends also told me just take it easy and not to be nervous.	Please record the first three family-related events that come to your mind today, and record them and the reasons why they happened. Here are some examples:<br>(1) My husband and I had dinner with our parents today.<br>(2) A good friend of mine got married today.
**4**	**My future**	Please recall 3 good things that happened to you today related to the future, and record them with the reasons why they happened. Here are some examples:<br>(1) Today, I saw other people's little kids, this time next year I can have my own baby!<br>(2) The weather forecast says the weather will be good, I'm looking forward to sunshine tomorrow.<br>(3) Tomorrow my favorite TV series will be updated.	Please record the first three future-related events that come to your mind today, and record them and the reasons why they happened. Here are some examples:<br>(1) We're going for a checkup tomorrow.<br>(2) Tomorrow my mother-in-law is coming to visit us.
**5**	**My life**	Please recall 3 good things that happened to you today related to your life, and record them with the reasons why they happened. Here are some examples:<br>(1) Today, I listened to my favorite songs, which gave me a lot of inspiration and memories.<br>(2) Today, I found a gentle sentence on my phone, which said, "I was born mediocre, and I was born proud. Let's be a happy ordinary person. "<br>(3) The emoticons my friend sent me today were so cute, I thought they were very interesting and funny.	Please record the first three life-related events that come to your mind today, and record them and the reasons why they happened. Here are some examples:<br>(1) I watch videos on the phone for an hour today.<br>(2) I watched a TV series with my husband this evening.

205 ***Evaluation and outcomes***

206 Primary outcomes will include exclusive breastfeeding and on-demand feeding during 207 postnatal hospitalization. Exclusive breastfeeding means that the baby is not given any

For peer review only - http://bmjopen.bmj.com/site/about/guidelines.xhtml

liquid or solid food other than breast milk. This indicator will be included in the postnatal questionnaire and also be double-checked in the hospital care records of formula. On-demand feeding means breastfeeding according to the baby's needs. Since the infant's early urination reflects breastfeeding, changing diapers 6-8 times a day in the early postpartum period is considered feeding on demand.

Secondary outcome indicators will mainly include other breastfeeding behaviors during postpartum hospitalization (first postnatal food for babies is breast milk; LATCH breastfeeding score), lactation-related hormone secretion (prolactin and IGF-I), and postpartum breastfeeding intention and postpartum emotion.

Data collection and follow-up

On the day of enrollment, pregnant women will be asked to complete an enrollment questionnaire providing information about general demographic characteristics and other factors influencing breastfeeding. Meanwhile, if the enrollment questionnaire finds prenatal depression among the participants, we will refer them to the department of psychology. Then the ecological instantaneous evaluation of both groups will be performed every week to determine the trend of change in intention and emotions until delivery. The measurement of emotion uses the Positive and Negative Affect Scale (PANAS) and 10 positive items for positive emotions proposed by Barbara L. Fredrickson for validation[24,25]. Postpartum questionnaires on breastfeeding behavior during postnatal hospitalization will be completed one week after delivery. Blood samples will be collected twice, after they are admitted to the hospital approaching delivery and after delivery at 7 to 8 a.m. (Vaginal Delivery: the morning after one night;

For peer review only - http://bmjopen.bmj.com/site/about/guidelines.xhtml

Cesarean section: the morning after two nights), and evaluated for prolactin and IGF-I to evaluate postpartum lactation ability. The blood draw will be conducted by nurses in the ward.

Upon recruitment, general demographic variables will be collected: education, occupation, number of permanent household members, total average annual household income, and expected date of birth. Other influencing factors will include mode of delivery, self-reported ease of breastfeeding, degree of sleep disturbance, self-reported importance of early exclusive breastfeeding, knowledge of breastfeeding, birth injury and nipple cracking pain scores, degree of hospital support, depression (measured by Edinburgh Postnatal Depression Scale, EPDS), anxiety (measured by Generalized Anxiety Disorder, GAD-7), breastfeeding intentions (measured by Infant Feeding Intentions Scale, IFI), breastfeeding behavior score (LATCH), resilience (Brief Resilience Scale, BRS), and potential number of traumatic events[26,27(p7),28–31]. Generally, Baby-friendly hospitals will take measures such as early sucking and skin-to-skin contact within 1hr after delivery, limitation of bottle used, room-in, etc[32]. We will also record and report routine measures taken in this Baby-Friendly hospital.

All questionnaires will be made through the questionnaire star website and distributed via WeChat. The corresponding data are stored in the questionnaire star account. Hormone test results will be recorded and saved manually. We will keep the personal information and data of each participant strictly confidential.

Data analysis

For peer review only - http://bmjopen.bmj.com/site/about/guidelines.xhtml

Descriptive analyses will be performed to show the baseline characteristics of the participants. To assess the intervention effects over time, MANOVA of repeated measuring will be taken for the change of positive and negative emotions, intention to breastfeed, and resilience trend across the weeks. To test the association among positive/negative emotions, lactation-related hormones, and breastfeeding behaviors, we will perform multiple linear regression or logistic regression in between and exploratory mediation analysis. To evaluate the effect of the intervention, we will take a two-sample T-test or Wilcoxon rank-sum test for quantitative data and a Chi-square test for qualitative data.

Data monitoring

The data monitoring committee is composed of members of the Science and Education Department of the hospital. As the administrative department of the hospital, the Ministry of Science and Education is in charge of scientific research, and reviews the integrity of scientific research to ensure the authenticity of data.

Patient and Public Involvement

The development of the research question and outcome measures isn't influenced by patients' priorities, experiences, and preferences. Participants and the public don't involve in the design, recruitment or conduct of the study. We will disseminate the results to study participants through subsequent publications. For this RCT, the burden of the intervention is assessed by the patients themselves through the questionnaire.

Discussion

Our study has several strengths. First, as distinct from current interventions focusing on

For peer review only - http://bmjopen.bmj.com/site/about/guidelines.xhtml

knowledge improvement, our study attempts to promote the secretion of lactation-related hormones by elevating positive emotions, then breastfeeding behaviors. Second, most previous interventions promoting breastfeeding were set in the postpartum period. Considering the importance of early breastfeeding experiences, we moved the intervention period to the late stages of pregnancy. Third, earlier mixed educational and supportive interventions are difficult to evaluate and replicate precisely. Our study will use the typical psychological measures, “Three Good Things”, to boost positive emotions, which is feasible for individuals to carry out and can be easier to promote and popularize among pregnant women.

This study also has several limitations. First, due to the restrictions of the testing technology and the intricacies involved in distinguishing between endogenous and exogenous sources, we will not test for oxytocin in the serum, although this has been widely reported to be associated with lactation. However, oxytocin release is related to prolactin levels, which will be included in our test[33]. Second, we limit the outcome measurement of breastfeeding-related behaviors to the postpartum hospitalization period, without following a 6-month or longer period. Nevertheless, exclusive breastfeeding in the early postpartum period (1 hour and within a few days after delivery) has been positively correlated with the duration of breastfeeding after delivery.

In conclusion, this study will test the effect of “Three Good Things” intervention not only on the emotions of women during pregnancy, but also on the secretion of colostrum and breastfeeding behaviors as well. This study will also help us further identify the physiological mechanisms of the association between emotions and

For peer review only - http://bmjopen.bmj.com/site/about/guidelines.xhtml

breastfeeding behaviors.

List of abbreviations

WHO	(World Health Organization)
UNICEF	(United Nations Children's Fund)
IGF-I	(Insulin-like growth factor Ⅰ)
PRL	(Prolactin)
PANAS	(Positive and Negative Affect Scale)
EPDS	(Edinburgh Postnatal Depression Scale)
GAD-7	(Generalized Anxiety Disorder-7)
IFI	(Infant Feeding Intentions Scale)
BRS	(Brief Resilience Scale)

Ethics and dissemination

The study has been approved by the Ethics Committees of the Women's Hospital School of Medicine at Zhejiang University (20190088) and the Wuyi First People's Hospital. The study will be conducted by the Declaration of Helsinki ethical principles for research involving human subjects. Results will be disseminated through peer-reviewed journals or international academic conferences. Written consent will be obtained from all participants.

Footnotes

Availability of data and materials

Not applicable.

Authors' contributions

KCX, YX, XJH and YY contributed to data acquisition and analysis. KCX and YX were in charge of the writing. SQL and SLW participated in the implementation of the

For peer review only - http://bmjopen.bmj.com/site/about/guidelines.xhtml

pilot trial. QY and QZ contributed to study design and manuscript revision. All authors approved the final version of the manuscript.

Funding statement

This work was supported by [Medical Scientific Research Foundation of Zhejiang Province, China] grant number [2021ZH054], [the National Natural Science Foundation of China] grant number [71974170]. The funders have no influences on study methods, analysis, interpretation, or publication of results.

Competing interests statement

The authors declare no conflict of interest.

Patient consent for publication

Not required.

Acknowledgements

We appreciate the support of the clinical staff of Zhejiang University School of Medicine and Wuyi First People's Hospital, including Xinyi Zhou, Suqing Liu, Xiaohong Qin, Qin Geng, and Di Gu.

Figure 1. Flow chart of the breastfeeding intervention project.The participants will be randomly divided into intervention group and control group. In phase T0, participants will complete the first questionnaire, including breastfeed intention, emotional scale, and writing practice persistence. In the T1 stage, the participants will complete a series of intervention questionnaires, including breastfeed intention, emotional scale, writing practice persistence. First lactation hormone test will be performed before delivery. The second lactation hormone test will be performed after delivery. Finally, a postpartum questionnaire will be performed about the outcome of lactation behavior.

References

For peer review only - http://bmjopen.bmj.com/site/about/guidelines.xhtml

1. Infant and young child feeding. Accessed December 27, 2021. https://www.who.int/en/news-room/fact-sheets/detail/infant-and-young-child-feeding
2. Andreas NJ, Kampmann B, Mehring Le-Doare K. Human breast milk: A review on its composition and bioactivity. *Early Hum Dev*. 2015;91(11):629-635. doi:10.1016/j.earlhumdev.2015.08.013
3. Johnson TJ, Patel AL, Bigger HR, Engstrom JL, Meier PP. Economic benefits and costs of human milk feedings: a strategy to reduce the risk of prematurity-related morbidities in very-low-birth-weight infants. *Adv Nutr Bethesda Md*. 2014;5(2):207-212. doi:10.3945/an.113.004788
4. Le Doare K, Holder B, Bassett A, Pannaraj PS. Mother's Milk: A Purposeful Contribution to the Development of the Infant Microbiota and Immunity. *Front Immunol*. 2018;9:361. doi:10.3389/fimmu.2018.00361
5. Lyons KE, Ryan CA, Dempsey EM, Ross RP, Stanton C. Breast Milk, a Source of Beneficial Microbes and Associated Benefits for Infant Health. *Nutrients*. 2020;12(4):E1039. doi:10.3390/nu12041039
6. The Lancet Breastfeeding Series papers . 2016.
7. Global strategy for infant and young child feeding,WHO.
8. Babies and mothers worldwide failed by lack of investment in breastfeeding. Accessed December 27, 2021. https://www.who.int/news/item/01-08-2017-babies-and-mothers-worldwide-failed-by-lack-of-investment-in-breastfeeding
9. Amiel Castro RT, Glover V, Ehlert U, O'Connor TG. Antenatal psychological and socioeconomic predictors of breastfeeding in a large community sample. *Early Hum Dev*. 2017;110:50-56. doi:10.1016/j.earlhumdev.2017.04.010
10. van Dellen SA, Wisse B, Mobach MP, Dijkstra A. The effect of a breastfeeding support programme on breastfeeding duration and exclusivity: a quasi-experiment. *BMC Public Health*. 2019;19(1):993. doi:10.1186/s12889-019-7331-y
11. Ystrom E. Breastfeeding cessation and symptoms of anxiety and depression: a longitudinal cohort study. *BMC Pregnancy Childbirth*. 2012;12:36. doi:10.1186/1471-2393-12-36
12. Seimyr L, Edhborg M, Lundh W, Sjögren B. In the shadow of maternal depressed mood: experiences of parenthood during the first year after childbirth. *J Psychosom Obstet Gynaecol*. 2004;25(1):23-34. doi:10.1080/01674820410001737414
13. Kehler HL, Chaput KH, Tough SC. Risk factors for cessation of breastfeeding prior to six months postpartum among a community sample of women in Calgary, Alberta. *Can J Public Health Rev Can Sante Publique*. 2009;100(5):376-380.
14. Emerson JA, Tol W, Caulfield LE, Doocy S. Maternal Psychological Distress and Perceived Impact on Child Feeding Practices in South Kivu, DR Congo. *Food Nutr Bull*. 2017;38(3):319-337. doi:10.1177/0379572117714385
15. Kitsantas P, Gaffney KF, Nirmalraj L, Sari M. The influence of maternal life stressors on breastfeeding outcomes: a US population-based study. *J Matern-Fetal Neonatal Med Off J Eur Assoc Perinat Med Fed Asia Ocean Perinat Soc Int Soc Perinat Obstet*. 2019;32(11):1869-1873. doi:10.1080/14767058.2017.1421929
16. Ritchie-Ewing G, Mitchell AM, Christian LM. Associations of Maternal Beliefs and Distress in Pregnancy and Postpartum With Breastfeeding Initiation and Early

For peer review only - http://bmjopen.bmj.com/site/about/guidelines.xhtml

Cessation. *J Hum Lact Off J Int Lact Consult Assoc*. 2019;35(1):49-58. doi:10.1177/0890334418767832
17. Corno G, Espinoza M, Maria Baños R. A narrative review of positive psychology interventions for women during the perinatal period. *J Obstet Gynaecol J Inst Obstet Gynaecol*. 2019;39(7):889-895. doi:10.1080/01443615.2019.1581735
18. Shariat M, Abedinia N. The Effect of Psychological Intervention on Mother-Infant Bonding and Breastfeeding. *Iran J Neonatol*. 2017;8(1):7-15. doi:10.22038/ijn.2017.16673.1191
19. Seligman MEP, Steen TA, Park N, Peterson C. Positive psychology progress: empirical validation of interventions. *Am Psychol*. 2005;60(5):410-421. doi:10.1037/0003-066X.60.5.410
20. Beanlands H, McCay E, Fredericks S, et al. Decreasing stress and supporting emotional well-being among senior nursing students: A pilot test of an evidence-based intervention. *Nurse Educ Today*. 2019;76:222-227. doi:10.1016/j.nedt.2019.02.009
21. Turner RA, Altemus M, Yip DN, et al. Effects of emotion on oxytocin, prolactin, and ACTH in women. *Stress Amst Neth*. 2002;5(4):269-276. doi:10.1080/1025389021000037586-1
22. Nicholas L, Dawkins K, Golden RN. Psychoneuroendocrinology of depression. Prolactin. *Psychiatr Clin North Am*. 1998;21(2):341-358. doi:10.1016/s0193-953x(05)70009-5
23. Field T. Prenatal anxiety effects: A review. *Infant Behav Dev*. 2017;49:120-128. doi:10.1016/j.infbeh.2017.08.008
24. Watson D, Clark LA, Tellegen A. Development and validation of brief measures of positive and negative affect: the PANAS scales. *J Pers Soc Psychol*. 1988;54(6):1063-1070. doi:10.1037//0022-3514.54.6.1063
25. Fredrickson BL. Positive Emotions Broaden and Build. In: Devine P, Plant A, eds. *Advances in Experimental Social Psychology, Vol 47*. Vol 47. Elsevier Academic Press Inc; 2013:1-53. doi:10.1016/B978-0-12-407236-7.00001-2
26. Cox JL, Holden JM, Sagovsky R. Detection of postnatal depression. Development of the 10-item Edinburgh Postnatal Depression Scale. *Br J Psychiatry J Ment Sci*. 1987;150:782-786. doi:10.1192/bjp.150.6.782
27. Spitzer RL, Kroenke K, Williams JBW, Löwe B. A brief measure for assessing generalized anxiety disorder: the GAD-7. *Arch Intern Med*. 2006;166(10):1092-1097. doi:10.1001/archinte.166.10.1092
28. Nommsen-Rivers LA, Dewey KG. Development and validation of the infant feeding intentions scale. *Matern Child Health J*. 2009;13(3):334-342. doi:10.1007/s10995-008-0356-y
29. Jensen D, Wallace S, Kelsay P. LATCH: a breastfeeding charting system and documentation tool. *J Obstet Gynecol Neonatal Nurs JOGNN*. 1994;23(1):27-32. doi:10.1111/j.1552-6909.1994.tb01847.x
30. Smith BW, Dalen J, Wiggins K, Tooley E, Christopher P, Bernard J. The brief resilience scale: assessing the ability to bounce back. *Int J Behav Med*. 2008;15(3):194-200. doi:10.1080/10705500802222972
31. Peterson C, Park N, Pole N, D'Andrea W, Seligman MEP. Strengths of character

For peer review only

For peer review only - http://bmjopen.bmj.com/site/about/guidelines.xhtml

and posttraumatic growth. *J Trauma Stress*. 2008;21(2):214-217. doi:10.1002/jts.20332

32. Li L, Song H, Zhang Y, et al. Breastfeeding Supportive Services in Baby-Friendly Hospitals Positively Influenced Exclusive Breastfeeding Practice at Hospitalization Discharge and Six Months Postpartum. *Int J Environ Res Public Health*. 2021;18(21):11430. doi:10.3390/ijerph182111430

33. Uvnäs Moberg K, Ekström-Bergström A, Buckley S, et al. Maternal plasma levels of oxytocin during breastfeeding-A systematic review. *PloS One*. 2020;15(8):e0235806. doi:10.1371/journal.pone.0235806

For peer review only

For peer review only - http://bmjopen.bmj.com/site/about/guidelines.xhtml

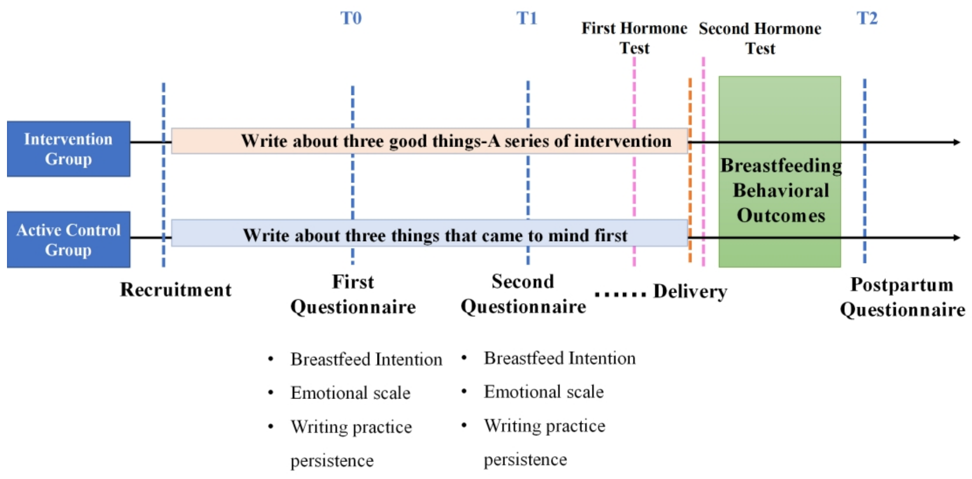

338x190mm (192 x 192 DPI)

STANDARD PROTOCOL ITEMS: RECOMMENDATIONS FOR INTERVENTIONAL TRIALS

SPIRIT 2013 Checklist: Recommended items to address in a clinical trial protocol and related documents*

Section/item	ItemNo	Description	
**Administrative information**			
Title	1	Descriptive title identifying the study design, population, interventions, and, if applicable, trial acronym	Title, Page 1
Trial registration	2a	Trial identifier and registry name. If not yet registered, name of intended registry	Trial registration, Page 2
	2b	All items from the World Health Organization Trial Registration Data Set	Trial registration, Page 2
Protocol version	3	Date and version identifier	NA
Funding	4	Sources and types of financial, material, and other support	Funding, Page14
Roles and responsibilities	5a	Names, affiliations, and roles of protocol contributors	Authors' contributions, Page14
	5b	Name and contact information for the trial sponsor	Correspondence, Page1
	5c	Role of study sponsor and funders, if any, in study design; collection, management, analysis, and interpretation of data; writing of the report; and the decision to submit the report for publication, including whether they will have ultimate authority over any of these activities	Competing interests, Page14
	5d	Composition, roles, and responsibilities of the coordinating centre, steering committee, endpoint adjudication committee, data management team, and other individuals or groups overseeing the trial, if applicable (see Item 21a for data monitoring committee)	NA
**Introduction**			
Background and rationale	6a	Description of research question and justification for undertaking the trial, including summary of relevant studies (published and unpublished) examining benefits and harms for each intervention	Background, paragraph 2-3
	6b	Explanation for choice of comparators	NA

For peer review only

Objectives	7	Specific objectives or hypotheses	Background, paragraph 4
Trial design	8	Description of trial design including type of trial (eg, parallel group, crossover, factorial, single group), allocation ratio, and framework (eg, superiority, equivalence, noninferiority, exploratory)	Methods/Design, paragraph 4

Methods: Participants, interventions, and outcomes

Study setting	9	Description of study settings (eg, community clinic, academic hospital) and list of countries where data will be collected. Reference to where list of study sites can be obtained	Study Design, Page 7-8
Eligibility criteria	10	Inclusion and exclusion criteria for participants. If applicable, eligibility criteria for study centres and individuals who will perform the interventions (eg, surgeons, psychotherapists)	Recruitment, Page 8
Interventions	11a	Interventions for each group with sufficient detail to allow replication, including how and when they will be administered	Intervention, Page 9-10
	11b	Criteria for discontinuing or modifying allocated interventions for a given trial participant (eg, drug dose change in response to harms, participant request, or improving/worsening disease)	NA
	11c	Strategies to improve adherence to intervention protocols, and any procedures for monitoring adherence (eg, drug tablet return, laboratory tests)	Intervention, Page 9-10
	11d	Relevant concomitant care and interventions that are permitted or prohibited during the trial	NA
Outcomes	12	Primary, secondary, and other outcomes, including the specific measurement variable (eg, systolic blood pressure), analysis metric (eg, change from baseline, final value, time to event), method of aggregation (eg, median, proportion), and time point for each outcome. Explanation of the clinical relevance of chosen efficacy and harm outcomes is strongly recommended	Evaluation and outcomes, Page 10
Participant timeline	13	Time schedule of enrolment, interventions (including any run-ins and washouts), assessments, and visits for participants. A schematic diagram is highly recommended (see Figure)	Data collection and follow-up, Page 10-11

For peer review only - http://bmjopen.bmj.com/site/about/guidelines.xhtml

Sample size	14	Estimated number of participants needed to achieve study objectives and how it was determined, including clinical and statistical assumptions supporting any sample size calculations	Sample size, Page 9
Recruitment	15	Strategies for achieving adequate participant enrolment to reach target sample size	Randomization and blinding, Page 9

Methods: Assignment of interventions (for controlled trials)

Allocation:

Sequence generation	16a	Method of generating the allocation sequence (eg, computer-generated random numbers), and list of any factors for stratification. To reduce predictability of a random sequence, details of any planned restriction (eg, blocking) should be provided in a separate document that is unavailable to those who enrol participants or assign interventions	Randomization and blinding, Page 9
Allocation concealment mechanism	16b	Mechanism of implementing the allocation sequence (eg, central telephone; sequentially numbered, opaque, sealed envelopes), describing any steps to conceal the sequence until interventions are assigned	Randomization and blinding, Page 9
Implementation	16c	Who will generate the allocation sequence, who will enrol participants, and who will assign participants to interventions	Randomization and blinding, Page 9
Blinding (masking)	17a	Who will be blinded after assignment to interventions (eg, trial participants, care providers, outcome assessors, data analysts), and how	Randomization and blinding, Page 9
	17b	If blinded, circumstances under which unblinding is permissible, and procedure for revealing a participant's allocated intervention during the trial	NA

Methods: Data collection, management, and analysis

Data collection methods	18a	Plans for assessment and collection of outcome, baseline, and other trial data, including any related processes to promote data quality (eg, duplicate measurements, training of assessors) and a description of study instruments (eg, questionnaires, laboratory tests) along with their reliability and validity, if known. Reference to where data collection forms can be found, if not in the protocol	Data collection and follow-up, Page 10-11
	18b	Plans to promote participant retention and complete follow-up, including list of any outcome data to be collected for participants who discontinue or deviate from intervention protocols	Intervention, Page 9-10

For peer review only - http://bmjopen.bmj.com/site/about/guidelines.xhtml

Data management	19	Plans for data entry, coding, security, and storage, including any related processes to promote data quality (eg, double data entry; range checks for data values). Reference to where details of data management procedures can be found, if not in the protocol	Data collection and follow-up, Page 10-11
Statistical methods	20a	Statistical methods for analysing primary and secondary outcomes. Reference to where other details of the statistical analysis plan can be found, if not in the protocol	Data analysis, Page 11-12
	20b	Methods for any additional analyses (eg, subgroup and adjusted analyses)	NA
	20c	Definition of analysis population relating to protocol non-adherence (eg, as randomised analysis), and any statistical methods to handle missing data (eg, multiple imputation)	NA
**Methods: Monitoring**			
Data monitoring	21a	Composition of data monitoring committee (DMC); summary of its role and reporting structure; statement of whether it is independent from the sponsor and competing interests; and reference to where further details about its charter can be found, if not in the protocol. Alternatively, an explanation of why a DMC is not needed	Data monitoring, Page 12
	21b	Description of any interim analyses and stopping guidelines, including who will have access to these interim results and make the final decision to terminate the trial	NA
Harms	22	Plans for collecting, assessing, reporting, and managing solicited and spontaneously reported adverse events and other unintended effects of trial interventions or trial conduct	NA
Auditing	23	Frequency and procedures for auditing trial conduct, if any, and whether the process will be independent from investigators and the sponsor	NA
**Ethics and dissemination**			
Research ethics approval	24	Plans for seeking research ethics committee/institutional review board (REC/IRB) approval	Ethics approval and consent to participate, Page 14
Protocol amendments	25	Plans for communicating important protocol modifications (eg, changes to eligibility criteria, outcomes, analyses) to relevant parties (eg, investigators, REC/IRBs, trial participants, trial registries, journals, regulators)	NA

Consent or assent	26a	Who will obtain informed consent or assent from potential trial participants or authorised surrogates, and how (see Item 32)	Ethics approval and consent to participate, Page 14
	26b	Additional consent provisions for collection and use of participant data and biological specimens in ancillary studies, if applicable	NA
Confidentiality	27	How personal information about potential and enrolled participants will be collected, shared, and maintained in order to protect confidentiality before, during, and after the trial	Intervention, Data collection and follow-up, Page 9-11
Declaration of interests	28	Financial and other competing interests for principal investigators for the overall trial and each study site	Competing interests, Page 14
Access to data	29	Statement of who will have access to the final trial dataset, and disclosure of contractual agreements that limit such access for investigators	NA
Ancillary and post-trial care	30	Provisions, if any, for ancillary and post-trial care, and for compensation to those who suffer harm from trial participation	NA
Dissemination policy	31a	Plans for investigators and sponsor to communicate trial results to participants, healthcare professionals, the public, and other relevant groups (eg, via publication, reporting in results databases, or other data sharing arrangements), including any publication restrictions	NA
	31b	Authorship eligibility guidelines and any intended use of professional writers	NA
	31c	Plans, if any, for granting public access to the full protocol, participant-level dataset, and statistical code	NA
**Appendices**			
Informed consent materials	32	Model consent form and other related documentation given to participants and authorised surrogates	Informed consent materials
Biological specimens	33	Plans for collection, laboratory evaluation, and storage of biological specimens for genetic or molecular analysis in the current trial and for future use in ancillary studies, if applicable	NA

*It is strongly recommended that this checklist be read in conjunction with the SPIRIT 2013 Explanation & Elaboration for important clarification on the items. Amendments to the protocol should be tracked and dated. The SPIRIT checklist is copyrighted by the SPIRIT

For peer review only - http://bmjopen.bmj.com/site/about/guidelines.xhtml

Group under the Creative Commons "Attribution-NonCommercial-NoDerivs 3.0 Unported" license.

For peer review only

For peer review only - http://bmjopen.bmj.com/site/about/guidelines.xhtml
